# Aphid herbivory on macrophytes drives adaptive evolution in an aquatic community via indirect effects

**DOI:** 10.1073/pnas.2502742122

**Published:** 2025-08-21

**Authors:** Martin Schäfer, Antonino Malacrinò, Christoph Walcher, Piet Spaak, Marie Serwaty-Sárazová, Silvana Käser, Thea Bulas, Christine Dambone-Bösch, Eric Dexter, Jürgen Hottinger, Laura Böttner, Christoph Vorburger, Dieter Ebert, Shuqing Xu

**Affiliations:** ^a^Institute of Organismic and Molecular Evolution, Johannes Gutenberg University Mainz, Mainz 55128, Germany; ^b^Institute for Evolution and Biodiversity, University of Münster, Münster 48149, Germany; ^c^Department of Biological Sciences, Clemson University, Clemson, SC 29634; ^d^Department of Agriculture, Università degli Studi Mediterranea di Reggio Calabria, Reggio Calabria 89122, Italy; ^e^Department of Aquatic Ecology, Swiss Federal Institute of Aquatic Science and Technology (Eawag), Dübendorf 8600, Switzerland; ^f^Department of Environmental Sciences, Zoology, University of Basel, Basel 4051, Switzerland; ^g^Institute of Integrative Biology, Eidgenössische Technische Hochschule Zürich, Zürich 8093, Switzerland; ^h^Institute for Quantitative and Computational Biosciences, Johannes-Gutenberg University of Mainz, Mainz 55128, Germany

**Keywords:** indirect ecological effects, experimental evolution, community ecology, adaptive evolution, plant-herbivore interaction

## Abstract

Indirect ecological effects occur when the impact of one species on another is mediated by a third species or the shared environment. Although indirect effects are ubiquitous in nature, we know remarkably little about how they may drive ecoevolutionary processes across community boundaries. Here, we show that insect (aphid) herbivory on macrophytes (duckweed) drove the adaptive evolution of a planktonic crustacean (*Daphnia magna*) in large outdoor aquatic mesocosms via indirect ecological effects. Aphid herbivory reduced duckweed growth and increased the nutrient and light availability in the water column, which promoted phytoplankton growth and boosted the abundance of *D. magna* that feed on phytoplankton. Whole-genome pool-sequencing and phenotypic assays revealed aphid-herbivory-mediated evolutionary changes to *Daphnia* population. Transplant experiments indicated that these evolutionary changes were adaptive. Furthermore, aphid-herbivory-mediated biotic and abiotic changes in the aquatic community increased the performance of the macrophytes and aphids. These results demonstrate that indirect ecological effects can shape ecoevolutionary interactions between seemingly independent species in natural communities.

## Significance

Indirect ecological interactions—where one species influences another via changes to a third species or the environment—are ubiquitous in natural communities but are rarely linked directly to rapid evolutionary change. Using large outdoor mesocosms, we show that terrestrial aphid herbivory on macrophytes (duckweed) cascades through species interaction networks to alter phytoplankton abundance and water chemistry, driving rapid adaptive evolution of the planktonic crustacean *Daphnia magna*. The aphid-herbivory-mediated changes in the aquatic community then benefited the aphids by enhancing their performance on duckweed. Our findings provide direct evidence that indirect ecological interactions in near-natural communities can drive rapid evolutionary changes and mediate ecoevolutionary interactions between seemingly independent organisms.

Indirect ecological interactions, which occur when one species impacts another through a third species or the environment, are ubiquitous in nature and can profoundly shape the dynamics of ecological communities (1). Indirect effects can significantly influence evolutionary processes and impact organism’s fitness in ways not immediately obvious in direct interactions like predation or competition (2). Theoretical models suggest that indirect effects can drive coevolution in mutualistic networks (3, 4) and that cascading trophic interactions can influence species fitness across ecosystem boundaries (5).

In many communities, ecological interactions drive rapid evolutionary changes that, in turn, alter these interactions (6, 7). For such ecoevolutionary dynamics, which are pivotal in shaping the complex interplay between species and their environments, indirect interactions can be critical. For example, Bassar et al. (8) demonstrated that adaptation to predators in fish populations had wide-reaching impacts on stream ecology, not only via their direct role as consumers but, more importantly, through indirect pathways such as nutrient recycling and trophic cascades. Additional modeling analysis of guppy populations suggested that even small indirect effects could radically change the process of adaptation (8). Given the complexity of natural ecosystems, it is important to experimentally assess how indirect interactions affect ecoevolutionary dynamics in order to fully understand how species evolve in natural communities.

**Fig. 1.**
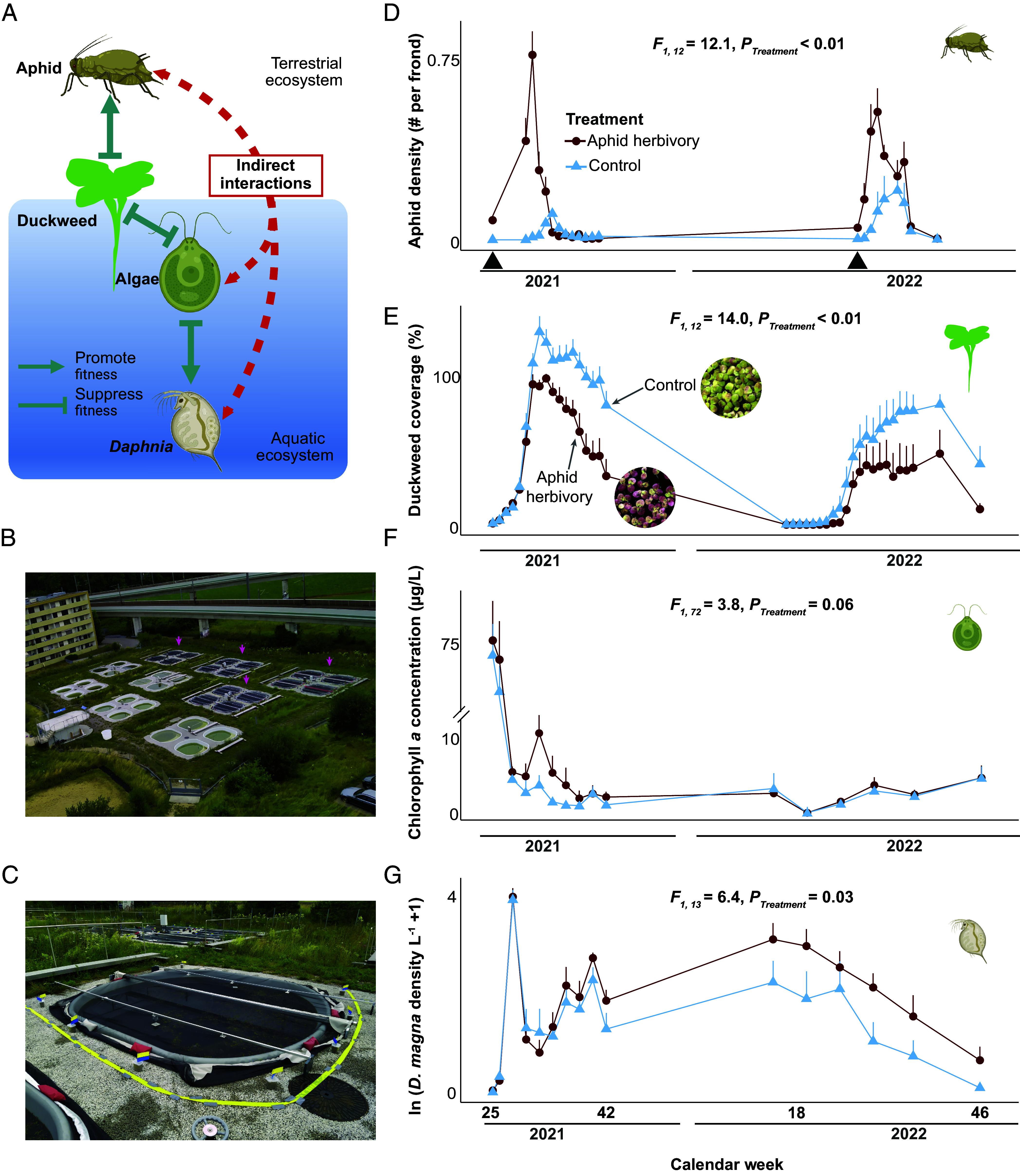
Aphid herbivory suppressed duckweed population growth and increased *Daphnia magna* abundance. (*A*) Simplified species interaction network in the community. (*B*) Overview of the outdoor experimental ponds. Arrows indicate the 4 × 4 ponds used for the experiments. (*C*) Close view of an experimental pond covered with a net to reduce aphid migration. (*D*–*G*) 2 y population dynamics for (*D*) aphid density, (*E*) duckweed (percentage of surface area covered), (*F*) total phytoplankton estimated by chlorophyll-a concentration, and (*G*) *D. magna* population density (in log scale). Duckweed surface coverage exceeds 100% when they grow in multilayers. *D*–*G*, Mean and SE are shown. Black arrows on the *x*-axis indicate aphid introductions into the herbivory ponds The X-axes mark experiment duration (in weeks & years). Dark red and light blue refer to herbivory and control treatment. Panel *E* contains pictures of the duckweed at calendar week 32 from the two treatments showing the marked color difference. Overview pictures of the ponds can be found in *SI Appendix*, Figs. S4–S12. *P*-values were estimated using linear mixed-effects models with time and pond block as random factors. Panel *A* and species icons are created with BioRender.com.

In shallow freshwater habitats, for example, floating macrophytes like *Spirodela polyrhiza* (giant duckweed) provide an interface for terrestrial insects (such as the waterlily aphid *Rhopalosiphum nymphaeae*), yet they simultaneously compete in the aquatic ecosystem with phytoplankton for nutrients and light (9) (Fig. 1*A*). The effect of a terrestrial herbivore on duckweed can, thus, cascade through the aquatic food web by altering duckweed abundance, nutrient availability, phytoplankton biomass, and, consequently, zooplankton populations (9). These cascading indirect effects may ultimately create new or altered natural selection on resident zooplankton and drive rapid evolutionary changes. Conversely, evolutionary or demographic changes in the zooplankton, together with changing nutrient dynamics in the aquatic community, could in turn influence the macrophyte’s growth, by shifting the relative competitive balance between macrophytes and phytoplankton.

Here, we investigate whether aphid herbivory on duckweed induces indirect ecological changes in a seminatural aquatic community (Fig. 1 *B* and *C*) and whether these changes drive the adaptive evolution of a keystone zooplankter, *D. magna*, a model organism that is feasible for experimental evolution in natural populations (10, 11). To test for feedback effects, we also ask whether changes in the aquatic community affect aphid–duckweed interactions. We hypothesize that indirect interactions mediate rapid ecoevolutionary dynamics between the aphid and *Daphnia*, two species that live in seemingly distinct communities.

## Results

### Aphid Herbivory Reduced Duckweed Growth and Altered the Aquatic Community.

We monitored the effects of aphid herbivory on the aquatic community over 2 y (2021 and 2022; *SI Appendix*, Fig. S1) in 16 outdoor experimental ponds where duckweed could grow in its natural environment. Aphids were introduced into half of the ponds, and we quantified aphid population size, duckweed growth, and changes to the plankton communities over two consecutive years. Aphid and duckweed populations grew exponentially in summer and declined in winter (Fig. 1 *D* and *E*, and *SI Appendix*, Fig. S2*A*) when the duckweed formed resting stages (turions) that sank to the bottom of the ponds. Given this lack of food resources and overwinter shelter, aphid populations declined to zero in late fall 2021 and were reintroduced back into the ponds in the spring of the second year (2022). By late summer of both years (between calendar weeks 30 and 36), aphid herbivory reduced the duckweed population size by ~30.7% in 2021 and by ~50.1% in 2022 (Fig. 1*E*). During the first year (2021), this reduction started shortly before the aphid population peaked (calendar week 30) and was still observable in spring 2022 when the duckweed grew back from its resting stages and before we reintroduced aphids into the ponds, suggesting that herbivory has a long-lasting impact on duckweed growth. In addition to reducing duckweed growth rate and population size, aphid herbivory also caused phenotypic changes in the duckweed, increasing the anthocyanin content, such as cyanidin-3-O-glycoside and cyanidin-3-O-(6-O-malonyl-beta-glucoside). This was likely responsible for the color differences observed during late summer, with the control group having predominantly greenish fronds, and the plants exposed to aphid herbivory having reddish fronds (Fig. 1 *E* *Insets* and *SI Appendix*, Figs. S3–S12).

Aphid herbivory on duckweed also caused significant changes in the aquatic community (*SI Appendix*, Table S1). The reduction of duckweed growth in 2021 was soon followed by an increase in phytoplankton (quantified by using chlorophyll-a concentration in the water as a proxy) between calendar weeks 30 and 35 (Fig. 1*F*). Phytoplankton composition was also quantified based on cell morphology in water samples taken during week 30 and 34 (*SI Appendix*, Fig. S2), which showed an increase not only in green algae and diatoms, such as Chlorophyta (*F_1, 13_* = 5.2, *P* = 0.04) and Bacillariophyceae (*F_1, 18_* = 11.4, *P* = 0.003), edible foods for *D. magna*, but also in cyanobacteria (*F_1, 35_* = 4.1, *P* = 0.05), a low-quality *D. magna* food. Shortly thereafter, an increase in *D. magna* populations, the main consumer of phytoplankton in the ponds, was observed. This increased *D. magna* population was likely the cause for the subsequent decline of phytoplankton in this treatment, which remained low until the end of the experiment in 2022, while the *Daphnia* populations remained high (Fig. 1*G*). From week 42 of the first year until the end of the experiment (over a year), the average *Daphnia* population size in the herbivory treatment approximately doubled compared to the control treatment (Fig. 1*G*). In addition to changes in the phytoplankton community, aphid herbivory also altered the abiotic environment in summer 2022, including increases in total phosphorus (71.5%) and N:P ratio (8.6%), water temperature (0.58 °C), and light availability underwater (3.4%) (*SI Appendix*, Figs. S13 and S14). Together, these results show that aphid herbivory on duckweed alters both the biotic and abiotic environment of the aquatic community, influencing the population size of *D. magna*.

### Aphid Herbivory Drove Rapid Adaptive Evolution of *D. magna*.

To test whether the abiotic and biotic shifts triggered by aphid herbivory also resulted in evolutionary changes in *D. magna*, we first monitored temporal changes in genotype frequencies. Specifically, we scored the strain-specific “adhesion” phenotype of the *Daphnia* parasite (*Pasteuria ramosa*), a Mendelian trait determined by alleles at well-characterized resistance loci (12–14). Because *D. magna* reproduces primarily clonally and these loci lie within large, highly polymorphic haplotypes, shifts in adhesion-phenotype frequencies sensitively track genotype frequencies that are caused by selection on linked genomic regions, as no parasite was found in the ponds. Adhesion phenotypes to three of the five *P. ramosa* strains (P15, P20, and P21)—used as genetic markers to infer the frequencies of the *D. magna* clones—differed significantly between control and herbivory ponds in both 2021 and 2022 (*SI Appendix*, Fig. S15), indicating that aphid herbivory changed *D. magna* genotype frequencies.

We then quantified genome-wide responses by pool-seq at the start (generation 0), mid-point (~6 generations), and end (~16 generations) of the experiment. Nucleotide diversity (θ_π_) did not differ between treatments (*F*-test, *P* > 0.1), but population divergence increased markedly: mean *F_ST_* rose from 0.04 ± 0.02 in 2021 to 0.1 ± 0.06 in 2022 (*SI Appendix*, Figs. S16 and S17), exceeding drift expectations (*SI Appendix*, Table S2). Concordantly, the fraction of SNPs with significant allele-frequency differences in Cochran–Mantel–Haenszel tests grew from 8.1% in 2021 to 28.6% in 2022 (*SI Appendix*, Fig. S18).

**Fig. 2.**
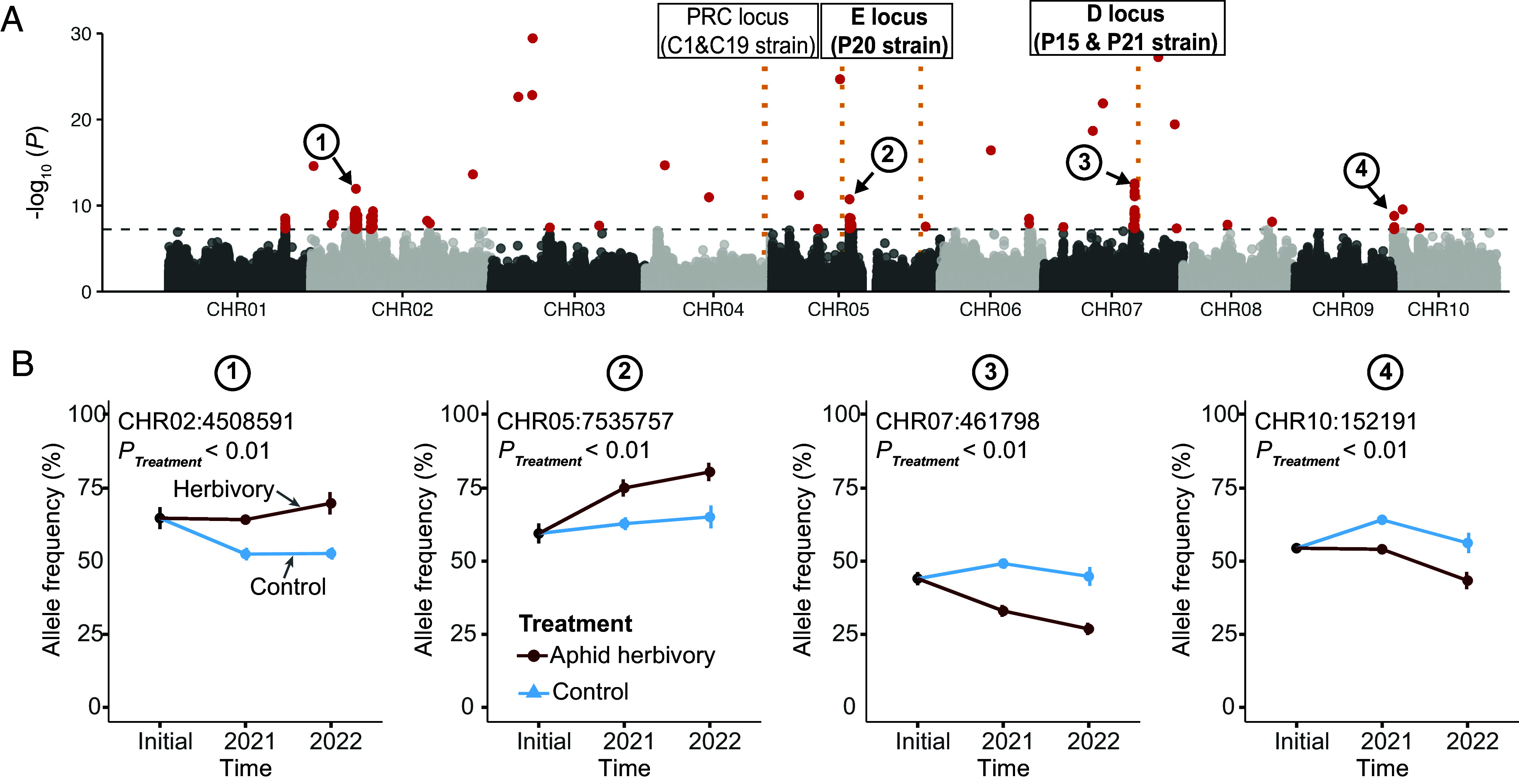
Aphid herbivory imposed genomic selection in *D. magna*. (*A*) Manhattan plot showing divergent selection imposed by aphid herbivory. *y*-axis shows the -log_10_ *P* value of each SNP based on the beta-binomial mixed-effects model (with time and pond block as random factors) between the two treatments. The dashed line refers to Bonferroni corrected *P* < 0.05 cutoff. Loci affecting the adhesion of *P. ramosa* strains are shown in black boxes. PRC locus affects strains C01 and C19; the E locus affects strain P20, and the D locus affects strains P15 and P21. *P. ramosa* strains that showed significant attachment phenotypes between control and aphid herbivory ponds are highlighted in bold. (*B*) Plots showing allele frequency changes of four representative SNPs labeled in *A*. Blue and dark red lines refer to control and aphid-herbivory treatments, respectively. Error bar refers to SE.

To identify the regions under divergent selection in the aphid herbivory ponds during those 2 y, we used the beta-binomial mixed-effects model (with year and pond block as random factors) to identify loci with different allele frequencies between control and aphid herbivory ponds. We found 141 SNPs (105 fell within 57 annotated genes) whose allele frequencies were significantly different (using a cutoff *P* < 0.05 after Bonferroni correction) between the two treatments (Fig. 2). Among them, 105 were in the genic region of 57 genes (*SI Appendix*, Table S3). Several of these were located at the beginning of chromosome 10, where significant signatures of selection were found only in the *D. magna* populations that evolved in herbivory ponds (*SI Appendix*, Figs. S18 and S19). Furthermore, several of the significantly changed SNPs were located close to loci known to affect strain-specific parasite attachment phenotype (14, 15), consistent with the observed changes in the parasite-adhesion phenotype (Fig. 2 and *SI Appendix*, Fig. S15).

**Fig. 3.**
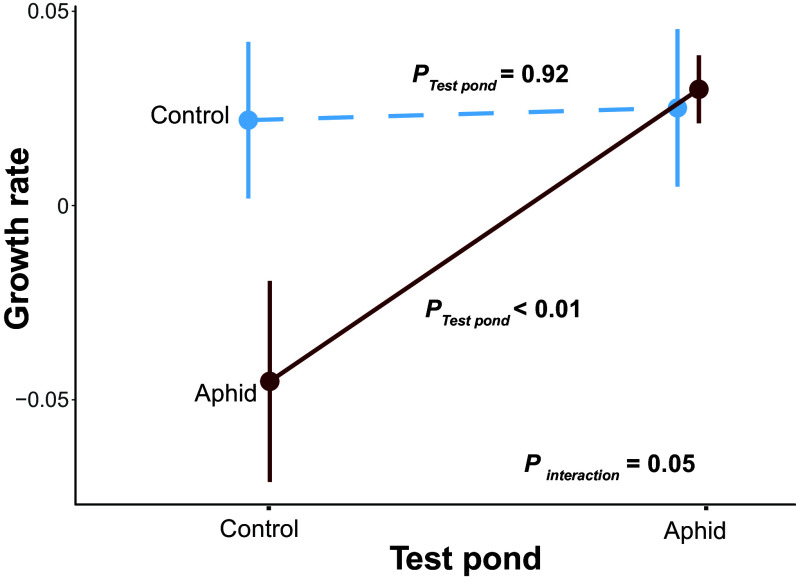
Evolution of *D. magna* is likely adaptive. The *x*-axis shows the environment of the transplant experiment (test pond). The *y*-axis refers to the per capita growth rate of *D. magna* (d^-1^). Light blue and dark red colored lines indicate the source community, i.e., the community in which the *Daphnia* evolved. *P*-values refer to the effects of the testing community. *P_interaction_* refers to the interaction effects between testing community and evolutionary treatment. Mean and SE are shown.

We further explored the adaptive nature of the observed evolutionary changes in *D. magna* with transplant experiments, conducted in the second year of the experiment (2022) (*SI Appendix*, Fig. S20). Using *D. magna* individuals collected from each pond, we placed them into both control and herbivory ponds, respectively, using PVC columns with mesh-covered cut-outs that allowed for an exchange of water and phytoplankton (*SI Appendix*, Fig. S20). This experiment was conducted once in spring (calendar week from 23 to 25) and once in summer (calendar week from 28 to 30). Both the season and aphid herbivory significantly affected *D. magna* growth rate. In summer, *D. magna* did not reproduce in most of the ponds, so their growth rates were similar between treatments. In spring, however, when *D. magna* grew rapidly in most ponds, the *D. magna* populations originating from aphid herbivory ponds showed significantly higher fitness when placed in aphid herbivory ponds compared to control ponds (*F_1,18_* = 14.4, *P* < 0.01, Fig. 3). The *D. magna* population that evolved in the control ponds had similar fitness in both the herbivore and control ponds (*F_1,15_* = 0.009, *P* = 0.9, Fig. 3).

Together, these data suggest that the evolution of *D. magna* populations in the aphid herbivory ponds reflects an adaptive response to this environment.

### Changes in the Aquatic Community Altered the Aphid–Duckweed Interaction.

**Fig. 4.**
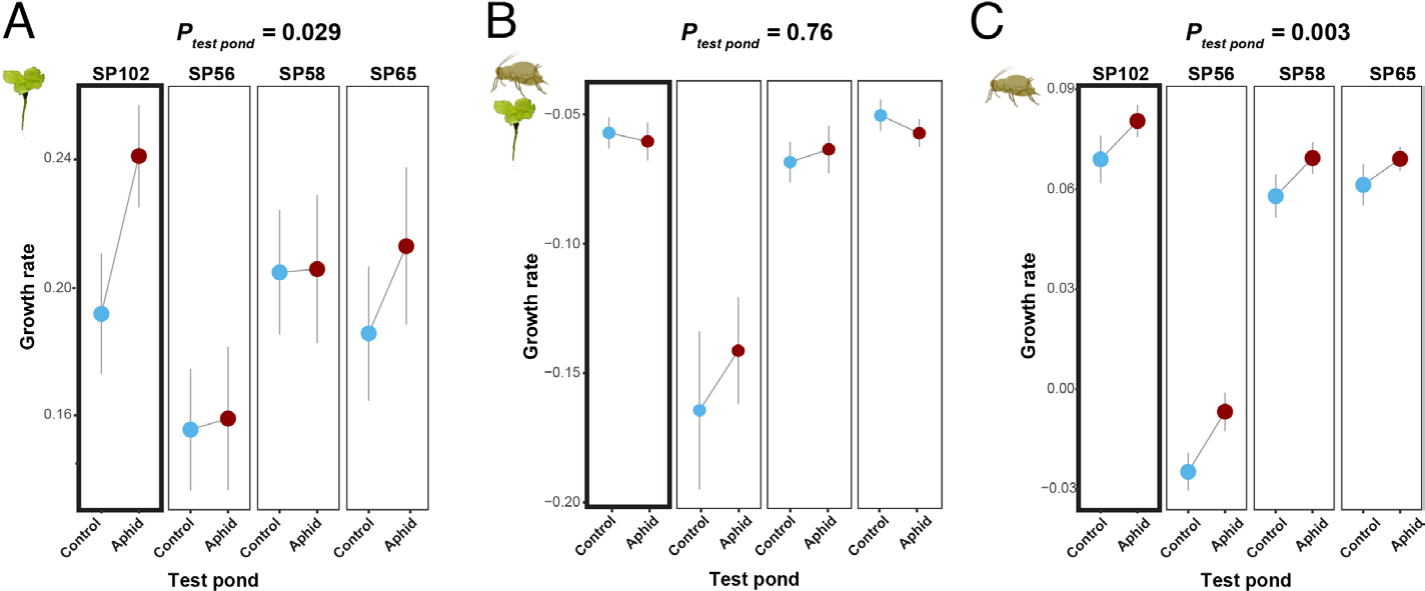
Evolution of the aquatic community altered aphid–duckweed interactions. The *x*-axis shows the test pond environment in which the transplant experiment was conducted. The *y*-axis refers to the per capita growth rate (d^-1^) of the duckweed without (*A*) or with (*B*) aphid herbivory. In panel (*C*) the *y*-axis refers to the aphid‘s growth rate. The average values from the two repeated experiments are shown. Light blue and dark red dots indicate the test pond where the plant and aphid fitness were quantified. *P*-values refer to the effects of the testing environment with experiment time as a random factor. All three parameters were measured for four duckweed genotypes collected in Europe (SP102, SP56, SP58, and SP65). Duckweed genotype SP102 was used in the mesocosm experiment (marked with a thick line box). Mean and SE are shown.

The changes caused by aphid herbivory in the aquatic community might, in turn, also alter aphid–duckweed interactions, leading to feedback effects. To test this hypothesis, we quantified duckweed growth rate and aphid resistance by growing aphids and duckweed inside the evolved aquatic communities (*SI Appendix*, Fig. S21), using four European duckweed genotypes to examine whether the response to the herbivory-influenced conditions in the ponds differed among duckweed genotypes and, thus, could drive duckweed evolution via the feedback effect. The experiments were conducted twice in 2022, once in late spring (from calendar week 20 to 25) and once in summer (from calendar week 25 to 30). While the four duckweed genotypes had different growth rates (*F_3, 87_* = 15.1, *P* = 5.5e-08, Fig. 4), the duckweed growth rate in the aphid ponds was higher than the one in control ponds (*F_1, 88_* = 4.9, *P* = 0.03, Fig. 4). The interaction effect between duckweed genotype and community was, however, nonsignificant (*F_3, 84_* = 1.4, *P* = 0.2), likely due to the small sample size. It is worth noting that the genotype we used to establish the initial population (SP102) appeared to show the highest growth rate increase in the aphid-herbivory environment. Among the four duckweed genotypes, aphid growth rates differed (*F_3, 95_* = 136.5, *P* < 0.001), suggesting that host plants varied in their defense levels or nutritional value. Interestingly, aphids grew faster in the aphid-herbivory ponds than in the control ponds (*F_1, 95_* = 9.3, *P* < 0.01), regardless of the plant genotype, suggesting that aphid-herbivory-induced community changes had positive feedback effects for aphids.

## Discussion

Indirect ecological effects are expected to play a crucial role in shaping community structure and influencing organismal fitness (3–5, 16). By experimentally manipulating insect herbivory on aquatic plants and measuring evolutionary responses in the planktonic crustacean *D. magna*, we demonstrate that indirect ecological effects can drive adaptive evolution in a natural multispecies community, shaping rapid multitrophic, ecoevolutionary dynamics. Here, aphid herbivory on duckweed shifted the competitive balance between duckweed and phytoplankton for light and nutrients in favor of phytoplankton, which in turn benefited the zooplankton.

The observed evolutionary changes in the large, genetically diverse *D. magna* population are likely due to a combination of changes in the quality and quantity of their planktonic food. Phytoplankton abundance and composition reacted strongly to the herbivory in the first year (*SI Appendix*, Fig. S2), likely due to the increased light and nutrient availability and higher temperature in those aquatic environments (*SI Appendix*, Fig. S14). These changes, in particular, the increased abundance of cyanobacteria, a low-quality food that can even be toxic to *D. magna*, could affect the growth of *D. magna* in a genotype-dependent manner (17–19). Accordingly, transplant experiments suggested that *D. magna* evolved in the aphid herbivory ponds had lower fitness when placed in the control ponds. However, *D. magna* that evolved in the control community showed comparable fitness in both communities, indicating that there are costs in adapting to the aphid herbivory-induced changes, e.g., increased abundance of cyanobacteria in 2021 (Fig. 1*F* and *SI Appendix*, Fig. S2), which has been shown in *D. mendotae* (16).

We found that aphid herbivory in these aquatic communities increased growth rates of duckweed and aphids in 2022 (Fig. 4 *A* and *C*), suggesting a potential feedback effect via indirect interactions in the community (20). These elevated growth rates are likely due to the increased availability of nutrients such as phosphate and the N:P ratio in the aphid ponds in 2022 (*SI Appendix*, Fig. S13). While it is likely that the increased nutrients in the aphid ponds are due to the reduced duckweed population size, the *D. magna* population was also considerably higher in the aphid ponds than the control ponds, suppressing algal growth (*SI Appendix*, Fig. S2) and thus possibly indirectly boosting the nutrient uptake competitiveness of the duckweed and food quality of the aphids.

Our experiment used only one aphid and one duckweed genotype. Under natural conditions, genetic variation exists for these species and can thus also evolve in response to direct and indirect interactions in the community (21, 22). Although the feedback effects mediated by these indirect interactions were not statistically different among the tested genotypes (likely due to a small sample size and number of analyzed genotypes), the strongest effects appeared in the duckweed genotype used to establish the initial pond community (Fig. 4), suggesting possible genotype‐specific responses. Taken together, our results suggest a plausible pathway for the coevolution of species across terrestrial and aquatic habitats through indirect interactions mediated by eco-evolutionary dynamics.

## Materials and Methods

### Establishing Outdoor Experimental Ponds.

In 2021, we established 16 outdoor experimental ponds at the Swiss Federal Institute of Aquatic Science and Technology (Eawag) in Dübendorf, Switzerland, using a similar setup as reported by Narwani et al. (23) (*SI Appendix*, Figs. S1 and S21). Each pond was approximately 4 m × 4 m, with a maximum depth of 1.5 m and a water volume of ~15 m^3^. As a first step, the ponds were cleaned with a high-pressure cleaner (*SI Appendix*, Fig. S22*D*). Then, we added 220 L of leaf litter collected from a nearby forest (next to Wangnerwald, 47°24′33.5″ N, 8°40′05.5″ E) to each pond to provide nutrients and shelter for different aquatic organisms (*SI Appendix*, Fig. S22*E*). The ponds were filled with tap water (calendar week 22) and inoculated with an initial plankton and microbe community collected from Lake Greifensee (47°20′59.592″ N, 8°40′40.736″ E) near the experimental site (calendar week 24). To each pond, we added 27 L of lake water collected from 5 to 6 m depth, first removing large organisms with a net of pore size 95 µm. Additionally, we added 2.8 L of concentrated plankton community (30 to 95 µm fraction) obtained by filtering approximately 1,750 L of lake water collected by sampling water columns from 0 to 10 m depth with a 30-µm mesh (*SI Appendix*, Fig. S22*F*) and pouring the concentrate through a 95-µm mesh to exclude larger organisms. Since our focal plant species, *Spirodela polyrhiza*, is known to produce turions (resting stages) in low phosphate environments (24), we additionally added 40.8 g of KH_2_PO_4_ per pond (ca. 20 µM; calendar week 24).

### Establishing the Aphid–Duckweed–Daphnia Populations in the Ponds.

In calendar week 25 of 2021, we added into each pond approximately 1,300 *D. magna* individuals from a mix of 122 genotypes (= clones, *SI Appendix*, Fig. S22*J*), additional algae (*Tetradesmus* spp. and *Nanochloropsis* spp.) used to feed *D. magna* in the lab, and approximately 5,000 *Spirodela polyrhiza* fronds from a single genotype (*SI Appendix*, Figs. S22*G* and S23; genotype SP102, originally collected in Switzerland and precultivated in the lab before the field experiment) covering approximately 1% of the water surface. We also added 18 great pond snails (*Lymnaea stagnalis*, *SI Appendix*, Fig. S22*H*) into each pond, as previous experiments suggested that they are important stabilizers for freshwater communities, as they control the growth of macroalgae (25). All *D. magna* genotypes were originally collected in Lake Aegelsee near Frauenfeld (47°33.48′N, 8°51.66′ E), Switzerland (26). These clones were produced from wild-caught individuals in the years 2014 to 2019 and kept in the laboratory by clonal propagation.

All *D. magna* clones were propagated in 400-mL jars (6 jars per clone) with abundant food (green algae *Tetradesmus* sp.) at a 16:8 h light:dark cycle and a temperature of 20 °C. When all clones were at their approximate carrying capacity, we mixed them into four 60-L tanks, topped to a volume of 45 L. This procedure was replicated 4 times. Subsamples were counted, resulting in an estimated population size of 7,300 *Daphnia* per 60-L tank. The following day, the four tanks were transported to the field site where the *D. magna* populations were well mixed, and 20 subsamples of equal size (2 L volume) were collected from each tank, resulting in 20 samples of 8 L volume with an estimated 1,300 *D. magna* individuals. Sixteen subsamples were used to inoculate the 16 experimental ponds. The initial inoculum for each mesocosm was large enough to minimize possible stochastic effects, influencing SNP and resistance phenotype frequencies. The remaining four samples were frozen in liquid nitrogen as samples from timepoint-0 for later genomics analyses (see below). From the timepoint-0 samples, we also collected 150 *D. magna* individuals to estimate parasite attachment phenotype (see below).

We also introduced 200 waterlily aphids (*SI Appendix*, Fig. S22*I*, *Rhopalosiphum nymphaeae*) from a single genotype into eight ponds, keeping the other eight ponds free from aphids as a control. The aphid populations were developed from a single individual collected on the University of Münster campus (51°57′40.7″ N, 7°36′55.8″ E) in September 2020. The ponds were arranged in four blocks of four ponds, each containing two aphid ponds and two control ponds. The populations of aphid, duckweed, and plankton communities were randomized.

To minimize aphid migration between ponds via air or over ground, we installed pond covers made from mosquito nets and placed sticky yellow and blue flags (insect traps) and sticky tape on the ground between ponds (*SI Appendix*, Fig. S22*C*). Control ponds were regularly monitored for aphids, and any accidental invaders were manually removed as long as feasible.

The experiment was run over 2 y—two summers and the winter in between. Pond covers were removed over the winter to avoid snowfall damage and were reinstalled in March 2022 (calendar week 11). Since aphids do not overwinter in ponds, they were reintroduced in calendar week 25 of 2022 into the same ponds that had received aphids the year before

Due to an unexpected extinction (for unknown reasons) of pond snails in pond 6A in 2021, we removed this replicate from all analyses. We also excluded the 2022 data from ponds 1D and 3D (aphid herbivory treatment) because the aphid populations did not successfully establish in these two ponds in 2022.

### Monitoring Population Dynamics in the Experimental Ponds.

Duckweed growth was assessed based on on-site estimates of pond surface coverage. Values above 100% represent multilayered growth. Additionally, overview pictures of the ponds were taken at each time point with a camera (GoPro Hero 7 and Hero 9) attached to a long stick. Aphid abundance was calculated based on close-up pictures taken with a mobile phone from 8 random positions at each pond (*SI Appendix*, Fig. S22*C*). Within a subsection of four pictures per pond, all aphids and fronds were counted to calculate the number of aphids per frond.

Plankton population dynamics were monitored using minor modifications to a previously established protocol (23) (*SI Appendix*, Fig. S24). Briefly, we sampled three vertical profiles of the water column using a Leibold-sampler, which consists of a 180-cm-long PVC tube (5 cm diameter) that can be closed via a wire with a plug at the bottom (*SI Appendix*, Figs. S22*D* and S22*E*). From each pond, we collected 10 L of water per sample. From this, we took a 1 L subsample for nutrient analyses (*SI Appendix*, Fig. S22*F*) and 1 L for chlorophyll-a and phytoplankton analyses (*SI Appendix*, Fig. S22*G*). During this subsampling, zooplankton were held inside the beaker with a 150-µm mesh. The remaining water was filtered through a 150-µm mesh to collect the retained zooplankton from the 10 L pond water sample (*SI Appendix*, Fig. S22*H*).

### Monitoring Nutrients and Abiotic Environment in the Experimental Ponds.

Orthophosphate-phosphorus (PO_4_^3−^-P) was measured photometrically using an Agilent Cary 60 spectrophotometer after reaction to phosphorus molybdenum blue complex. Total phosphorous (P) was measured after decomposition by autoclaving at 121 °C with subsequent determination of PO_4_^3-^-P as above. Ammonium-nitrogen (NH_4_^+^-N) was measured photometrically using an Agilent Cary 60 spectrophotometer after reaction to a blue complex by Berthelot reaction. Total organic carbon (TOC) was measured on a Shimadzu TOC-L CSH after catalytic combustion at 720 °C with subsequent measurement of CO_2_ via an infrared detector.

Pond water pH and oxygen concentrations were monitored regularly using handheld sondes (Multi 3630 IDS with an FDO 925 oxygen sensor and a SenTix 940-3 pH sensor, all from WTW) (*SI Appendix*, Fig. S24*B*). The dipping probes were placed 75 to 80 cm below the water surface. To continuously analyze water temperature and light penetration, we deployed a logger in the middle of each pond 30 to 35 cm below the water surface (*SI Appendix*, Fig. S24*A*): six ponds (3 per treatment) received a temperature logger (HOBO pendant temperature 64 K, UA-001-64), and 10 ponds (5 per treatment) were outfitted with a temperature logger with a light sensor (HOBO Pendant Temperature + Light 64 K, UA-002-64). The data were recorded every 15 min. Temperature was expressed as weekly average temperature. Light data (in LUX) were summed up daily and averaged for each week.

### Assessing Parasite Attachment and Genomic Changes in the *D. magna* Populations.

At defined time points, *Daphnia* population samples were collected from the experimental ponds to assess parasite attachment and genomic changes using a pool-seq approach. Samples at timepoint 0 (June 22^nd^, 2021, calendar week 25) were collected from the original inoculum, as described above. For all later time points, samples were collected using handheld nets with a mesh width of about 0.2 mm, taking care to ensure that every depth and area of the ponds were swept. Samples were then brought to a laboratory, inspected using stereo microscopes, and sorted to include only *D. magna*. From each sample, about 250 adult *D. magna* were collected and frozen with a small amount of water in liquid nitrogen. Sampling dates for the pool-seq samples were September 22nd, 2021, (calendar week 38) and July 28th, 2022 (calendar week 30). The samples used to assess parasite attachment phenotypes were transported in 2-L bottles to the University of Basel laboratory, where 100 animals from each pond were placed individually in a 100-mL jar. Animals were tested for parasite resistance as described below. Sampling dates roughly coincided with the sampling dates for the pool-seq samples but were more spread out, because the workload associated with testing 16 times 100 animals was too high to be performed in a short time interval. Therefore, samples were taken across a time period of about 4 wk (from September 22nd to October 16th, 2021, and from July 3rd to July 28th, 2022). For each collection, samples were randomized (balancing treatments and blocks exactly) and assessed blindly for phenotype attachment with regard to the treatment.

For each clone, we assessed the attachment of five isolates of the common bacterial pathogen *Pasteuria ramosa* (27). In short, *D. magna* individuals were exposed to fluorescently labeled parasite endospores. After 20 to 60 min, animals were assayed with a fluorescence microscope to check for endospores attached to the fore or hindgut. The ability of the parasite to attach to the gut epithelium is a prerequisite for infection and correlates very strongly with infection success (27). The loci determining attachment are Mendelian but interact epistatically with each other (13, 14, 28). The diversity of our *D. magna* clone panel was enriched for rare attachment phenotypes.

For the pool-seq samples, we extracted DNA from pooled individuals from each pond using the CTAB method. The total DNA was sequenced with 150 bp paired-end reads at ~480X coverage for each sample using an Illumina NovaSeq 6000 instrument by Novogene in Beijing, China. Raw data were quality-checked and trimmed using TrimGalore v0.6.1 (29), and reads were mapped toward the *D. magna* reference genome (30) using BWA (31) and SAMtools (32). Signatures of selection in *D. magna* populations from both herbivory and control ponds were detected using CLEAR based on time-series data (33). We then used SAMtools (32), PoPoolation2 (34), and poolfstat (35) to estimate genome-wide *F_ST_* between and within treatments and sample time points, with a minimum read coverage of 20 and a maximum coverage of 400 as the cutoff. A principle component analysis was performed using the R-package “pcadapt” (36). A heatmap showing similarities among each pool was created using a pairwise *F_ST_* matrix estimated by poolfstat. We detected treatment-specific SNPs with the Cochran–Mantel–Haenszel (CMH) test (37, 38), as implemented in *PoPoolation2*, with Bonferroni‘s corrected *P*-value of 0.05 as the cutoff. For each SNP, the CMH procedure treats the allele-count table from every pond (replicate) as a separate 2 × 2 contingency table (treatment × allele) and then combines them into a single stratified χ^2^ statistic. This approach controls for variation among replicates while testing whether an allele shows a consistent, directionally identical frequency shift across all ponds, thereby distinguishing true selection signals from random drift.

To identify genomic regions that were under selection during the 2 y, we used the beta-binomial mixed-effects model [from R-package glmmTMB v1.1.9 (39)] with sampling time and pond block as random factors. Beta-binomial mixed-effects models are designed for proportion or count data that exhibit more variability (overdispersion) than can be expected using a simple binomial model. Genetic diversity ($\theta_{\pi }$) was estimated using grenedalf (40) with minimal and maximum read coverage of 20 and 400, respectively. The genomic positions of the resistance loci were identified by mapping the sequence from refs. 13–15 with the genome.

### Transplant Experiments.

To assess the effects of the aquatic community on *D. magna* growth, we used a 140-cm-long vertical PVC tube (5 cm diameter, *SI Appendix*, Fig. S20) closed at the bottom with four equally distributed openings (12 × 6 cm) covered with a 150-µm mesh to allow for water and nutrient exchange. At the beginning of the transplant experiments, we put 50 similar-sized *D. magna* females (without eggs) from the ponds into each tube, sorted them using a stereomicroscope and either kept them in their original pond, moved them to another pond from the same treatment, or moved them to a pond with a different treatment. At the end of the experiment, the *D. magna* inside each tube were collected on a 1 mm mesh, from which they were kept in 100% EtOH. Samples were stored in a 50-mL Kautex bottle at room temperature until further analysis. We counted *D. magna* individuals using a Leica M205 C stereomicroscope and performed the experiments twice. The first experiment lasted 13 d (between calendar week 23 and 25), and the second experiment lasted for 12 d (between calendar week 28 and 30). The per capita growth rate (per day) was calculated as ln(N_end_) - ln(N_start_), where N_end_ and N_start_, refer to the number of *D. magna* at the end and beginning of experiments, respectively, divided by the experiment durations.

To assess the effects of the aquatic environment on the fitness of the duckweed and aphid, we performed growth assays using the floating boxes with mesh (*SI Appendix*, Fig. S21) that allowed for the exchange of water and plankton while preventing the escape of the duckweed and aphids. The boxes were constructed from plastic boxes with lids (Eurobox, Auer Packaging, inside measures: w × d × h 17 × 12 × 11.5 cm). Holes were cut into the bottom (15.3 × 10.3 cm) and lid (16.8 × 11.8 cm) and covered with steel mesh (bottom: mesh size 0.63 mm, wire diameter 0.22 mm, material 1.4301 steel; lid: mesh size 0.14 mm, wire diameter 0.112 mm, material 1.4301 steel). To make it float, each box was fitted with an external styrodur frame.

Growth assays were performed with four duckweed genotypes (SP102, SP56, SP58, and SP65) originally collected from different areas in Europe (41). At the beginning of the experiment, we put approximately 20 duckweed individuals (fronds) from one of those genotypes into each floating box. For each pond and each genotype, we used two boxes: In one, we added five aphids, while the other one was left without aphids (control). Approximately 2 wk later, we collected both duckweeds and aphids, froze them in liquid nitrogen and measured their dry weight. To estimate the number of duckweed individuals per box, we collected and counted up to 100 duckweed individuals from each box, measured their dry weight, and then calculated dry weight per frond to estimate the total number of duckweed individuals for each box. We also used those fronds (up to 100) to estimate the number of aphids in each box, counting the number of aphids per frond and multiplying it by the total number of fronds per box. Growth rate (per day) was calculated as ln(N_end_) - ln(N_start_), where N_end_ and N_start_ refer to the number of duckweed or aphids at the end and beginning of experiments, respectively, divided by the number of days. Duckweed resistance was estimated by calculating the differences in growth rate between aphid-treated and control boxes for each genotype in each pond.

### Statistical Methods.

Ecological and phenotypic responses (duckweed coverage, aphid density, phytoplankton and zooplankton abundance, nutrient levels, temperature, and oxygen levels) were analyzed using linear mixed‐effects models according to this formula: lmer[Response~AphidTreatment*Year + poly(Week, 2) + (Time|PondID), REML = T)]. We used polynomial effects of the week factor because many changes are seasonal. The “bobyqa” method was used to control model fitting. For the population size of phytoplankton and zooplankton, we used natural log-transformed data. The parasite attachment data were analyzed using generalized linear mixed-effects models with the formula glmer[Response ~ AphidTreatment + (1 + PondID|PondBlock), family = binomial(link = “logit”)]. *Daphnia* transplant experiment data were analyzed using linear mixed-effects models with the formula GrowthRate ~ TreatmentInTest_Community+TreatmentInTest_Community: TreatmentInOrigin_Community+ (1 | PondBlock). The effects of transplanting *Daphnia* into different ponds but with the same treatment were initially included in the full model but later removed due to their insignificant effects. The duckweed transplant experiment data were analyzed using the formula lmer[GrowthRate ~ AphidTreatment + (1 | PondBlock)]. Aphid growth rate from the duckweed transplant experiments was analyzed using the formula lmer[GrowthRate ~ AphidTreatment +PlantGenotype + (1 | PondBlock)].

All linear mixed-effects models and generalized linear mixed-effects models were run using lme4 package (v 1.1-36). Model optimizations were performed by removing nonsignificant variables after comparing models. The *P*-values from all linear mixed‐effects models and model optimizations were determined by the *Anova* function from the “*car*” package (v3.1-3) using *F*-test on the linear mixed-effects models. The *P*-value and effects of the fixed effects are shown in *SI Appendix*, Table S1.

## Supplementary Material

Appendix 01 (PDF)

## Data Availability

Raw sequencing reads data have been deposited in SRA under the BioProject PRJNA849360 (42). Raw monitoring data and data analysis scripts for generating figures are deposited on GitHub (43) and Dryad (44).
