# Supplementary material for: Aphid herbivory on macrophytes drives adaptive evolution in an aquatic community via indirect effects
Source: Proc Natl Acad Sci U S A. 2025 Aug 21;122(34):e2502742122. doi: 10.1073/pnas.2502742122 (PMC12403121; doi:10.1073/pnas.2502742122)
Supplement: Supplementary file 1 — Appendix 01 (PDF) [file pnas.2502742122.sapp.pdf]

PNAS

Supporting Information for
Aphid herbivory on macrophytes drives adaptive evolution in an aquatic community via indirect effects

Martin Schäfer[1,2,†], Antonino Malacrinò[3,4,†], Christoph Walcher[5], Piet Spaak[5], Marie Serwaty-Sárazová[2], Silvana Käser[5], Thea Bulas[5], Christine Dambone-Bösch[5], Eric Dexter[6], Jürgen Hottinger[6], Laura Böttner[2], Christoph Vorburger[5,7,*], Dieter Ebert[6,*] and Shuqing Xu[1,2,*]
1 Institute of Organismic and Molecular Evolution (IomE), Johannes Gutenberg University Mainz, Mainz, Germany
2 Institute for Evolution and Biodiversity, University of Münster, Münster, Germany
3 Department of Biological Sciences, Clemson University, Clemson, SC, USA
4 Department of Agriculture, Università degli Studi Mediterranea di Reggio Calabria, Reggio Calabria, Italy
5 Swiss Federal Institute of Aquatic Science and Technology (Eawag), Dübendorf, Switzerland
6 Department of Environmental Sciences, Zoology, University of Basel, Basel, Switzerland
7 Institute of Integrative Biology, ETH Zürich, Zürich, Switzerland
† These authors contributed equally to this work
* Corresponding authors: shuqing.xu@uni-mainz.de; christoph.vorburger@eawag.ch; dieter.ebert@unibas.ch

This PDF file includes:

Supporting text
Figures S1 to S24
Tables S1 to S3

Supporting text

Additional results and discussions

Several of the identified significant SNPs were located close to loci known to affect strain-specific parasite attachment and resistance (13, 16) (Figure 2), such as within the large (nearly 5 MB) non-recombining haplotype that covers most of the right arm of Chromosome 5 (Figure 2 A and B), which determines the attachment of *P. ramosa* P20 strain to *D. magna*. Similarly, several significant SNPs were located on chromosome 7 near the D-locus (Figure 2 A and B), which determines the attachment ability of *P. ramosa* strain P15 and P21 to *D. magna*. However, none of the significant SNPs were found near the *Pasteuria* resistance complex (PRC) locus on chromosome 4 (previously called ABCFG locus), which affects the attachment of *P. ramosa* strains C1 and C19. These changes in allele frequency near the parasite-attachment locus are highly consistent with the phenotypic changes observed from the attachment tests (Figure S15).

According to our genomic analysis, aphid herbivory-imposed selection drove genomic changes in the *D. magna* populations (Figure 2). Genome-wide population differentiation between the control *D. magna* populations and the herbivory pond populations increased 2.4-fold in 2022 over 2021. Because *D. magna* sexual reproduction and recombination levels were limited during our experimental phase, we expected a large proportion of the allele frequency changes in the populations to be affected by clonal selection, during which the entire genome is in genetic linkage. Consistently, the phenotype frequencies of the parasite attachment genes, which we used as genetic markers, diverged among the treatments even though the parasite was not present in the ponds. All but one of these attachment genes are in large non-recombining regions in the *D. magna* genome (12, 13), suggesting that linked genes may have been affected by selection. These haplotype blocks are known to evolve very quickly in *D. magna* populations (21), so the change in parasite attachment phenotype frequency might have been a side-effect of selection on nearby genes, indicating that if parasites had been present, their interaction with the host could potentially have been impacted by the herbivory treatment as well. Alternatively, selection by the parasite on the host (21) might have influenced or even overridden the response of *D. magna* to the herbivory treatment and potentially other downstream effects.

Materials and Methods

Analysis of anthocyanin in duckweed populations

Samples from the duckweed populations in each pond were collected, cleaned from aphids and other large contaminants, washed with fresh tap water, dried using paper towels, and snap-frozen in liquid nitrogen. Samples were stored until further processing at -20 °C. Anthocyanins were analyzed by LC-MS and HPLC-PDA, similar to those described by Malacrinò et al. (1). In brief, approximately 10 mg of finely ground, freeze-dried plant tissue was extracted with acidified methanol. Fresh weight was estimated based on the fresh weight to dry weight ratio of each sample. Cyanidin-3-O-glucoside was analyzed by LC-MS. Before analysis, the sample extracts were diluted 1:100 in an aqueous mix of isotope-labeled amino acids (algal amino acid mixture-^{13}C-^{15}N; Sigma-Aldrich). Chromatographic separation was done on a Shimadzu Nexera X3 equipped with an Agilent 1290 infinity II inline filter (0.3 µm) and a ZORBAX RRHD Eclipse XDB-C18 column (3×50 mm, 1.8 µm; Agilent Technologies). Separation was done in gradient mode with 0.05 % formic acid (Fisher Chemical), 0.1% acetonitrile (Fisher Chemical) in water as Solvent A and methanol (Fisher Chemical) as Solvent B, with the column oven set to 42 °C and a flow rate of 500 µL/min using the following solvent settings (Time [min]/B[%]): 0.0/2, 1.5/2, 3.5/100, 4.5/100, 5.0/2, 6.0/2. The LC-system was coupled to a Shimadzu LCMS-8060 mass spectrometer equipped with an ESI source, which was operated in positive ionization mode and the following settings: Nebulizing Gas Flow: 3 L/min; Heating Gas Flow: 10 L/min; Drying Gas Flow: 10 L/min; Interface Temperature: 300 °C; DL Temperature 250 °C; Heat Block Temperature: 400 °C; CID Gas: 270 kPa; Q1 Resolution: Unit; Q3 Resolution: Unit. Analysis was done in the multi-reaction-monitoring (MRM) mode using the following precursor to product ion transitions: Cyanidin-3-O-glucoside (quantifier), 449.10 -> 137.10; Cyanidin-3-O-glucoside (qualifier), 449.10 -> 213.15; $^{13}C_9$, $^{15}N_1$-Phenylalanine, 176.11 -> 129.25. The Dwell time [ms], collision energy [V], Q1Pre Bias [V] and Q3 Pre Bias [V] were: 32/32/247, -54/-54/-15, -20/-20/-20 and -14/-22/-20, respectively for Cyanidin-3-O-glucoside (quantifier)/Cyanidin-3-O-glucoside (qualifier)/$^{13}C_9$, $^{15}N_1$-Phenylalanine. The retention time of Cyanidin-3-O-glucoside was 3.38 min,

and for $^{13}C_9$, $^{15}N_1$-Phenylalanine was 2.52 min. Relative quantification of Cyanidin-3-O-glucoside was done based on the $^{13}C_9$, $^{15}N_1$-Phenylalanine internal standard from the isotope-labeled amino acid mixture and after normalization to the fresh weight of extracted plant material.

For absolute quantification of Cyanidin-3-O-glucoside and additional analysis of Cyanidin-3-O-(6-O-malonyl-beta-glucoside) the pure sample extracts were further analyzed via HPLC-PDA, which was done on a Shimadzu Nexera XR equipped with a photodiode array detector, a Nucleodur Sphinx RP column (250×4.6 mm, 5 µm, Macherey-Nagel) and an EC 4/3 Nucleodur Sphinx RP pre-column (5 µm, Macherey-Nagel). Separation was done in gradient mode with 0.2% formic acid (Fisher Chemical), 0.1% acetonitrile (Fisher Chemical) in water as Solvent A and acetonitrile (Fisher Chemical) as Solvent B, with the column oven set to 20 °C and a flow rate of 1300 µL/min using following solvent settings (Time [min]/B[%]): 0.0/10, 8.0/21, 18.0/49, 18.1/100, 19.0/100, 19.1/10, 24.0/10. Measurement was performed with a PDA detector. Cyanidin-3-O-glucoside and Cyanidin-3-O-(6-O-malonyl-beta-glucoside) were analyzed at an absorption wavelength of 517 nm and their retention times of 7.055 min and 9.410 min, respectively. Quantification was done based on the comparison with the molar quantity of an external Cyanidin-3-O-glucoside standard curve and after normalization to the fresh weight of extracted plant material.

Quantify total phytoplankton abundance

We estimated the total phytoplankton abundance in the pond by measuring chlorophyll-*a* content in the water. To this end, each sample was filtered using a 25-mm GF/F filter on a 50-mL syringe. Samples were filtered until a noticeable backpressure occurred or the filter turned green. The volume of filtered water was noted for later normalization. Afterward, excess water was removed from the filters by pushing another syringe filled with air through the filter. Filters were then removed from the support and shortly dried on a paper towel before they were placed in aluminum-covered tubes on ice and subsequently stored at -20 °C until further processing. We extracted chlorophyll-a from the filters with 5 mL 90% EtOH after vortexing, sonication, and overnight incubation at 4 °C in the dark. Subsequently, the extracts were passed through 0.2-µm cellulose acetate syringe filters before they were analyzed on a Hitachi 2000UV/VIS Spectrophotometer LLG-uniSPEC4 based on the absorption at 750 nm and 665 nm and an external chlorophyll-a standard. The chlorophyll-a content was calculated using the following formula:

$$\frac{(E_{665} - E_{750} - E_{665cuvette}) * v}{CHL\ factor * V * d}$$

where E_{665} refers to extinction at 665 nm, E_{750} refers to extinction at 750 nm, $E_{665cuvette}$ refers to extinction at 665 nm of a cuvette filled with 90% EtOH, *v* refers to the volume of 90% EtOH, *CHL* factor refers to a correction factor (0.000082), *V* refers to the filtrated volume and *d* refers to the cuvette length (5 cm).

Determine the taxonomic composition of phytoplankton

To determine the taxonomic composition of phytoplankton, the water samples were preserved by adding 5 mL of Lugol's solution into 50 mL of pond water. Samples were stored at 4 °C until further processing. For analysis, subsamples were placed in a Utermöhl counting chamber for 24 hours. Chlorophyll-*a* concentrations were used as a guide for high-density samples, of which 3 mL were sedimented. Afterward, the tube was removed, a cover slide was placed on top of the counting chamber, and the sedimented plankton was analyzed under a Zeiss Axiovert 135 inverted microscope (2). To this end, phytoplankton were identified (mostly to genus level) and counted in 40 randomly chosen fields of view (out of 1,686) with 320× magnification and 40 fields of view (out of 6,900) with 640× magnification. If one taxon was highly abundant (>100 cells in 10 fields of view), we counted this taxon in only 10 fields of view.

The zooplankton collected from the 10 L of pond water via a 150-µm mesh was eluted from the mesh with 100 % EtOH each into a 50-mL Kautex bottle. Samples were stored at room temperature until further analyses. Samples were analyzed under a Leica M205 C stereomicroscope at 10-50X magnification, and counts were converted to individuals per liter of

pond water. Adult Cladocera were identified at the species level, juvenile Cladocera and copepods at the genus level, and other zooplankton at higher taxonomic levels.

Check the presence of *P. ramosa*

We used the Pool-seq data to test for the presence of *P. ramosa* in our samples. Using SAMtools, we selected the unmapped sequences. These data were then used as input for Kraken2 (3) to characterize the *Daphnia*-associated microbial community using a custom reference dataset that included the bacterial and fungal RefSeqs from NCBI and, additionally, the reference genome of *P. ramosa* (4). This analysis did not identify any parasite sequence in any of our samples.

Supplementary figures and tables

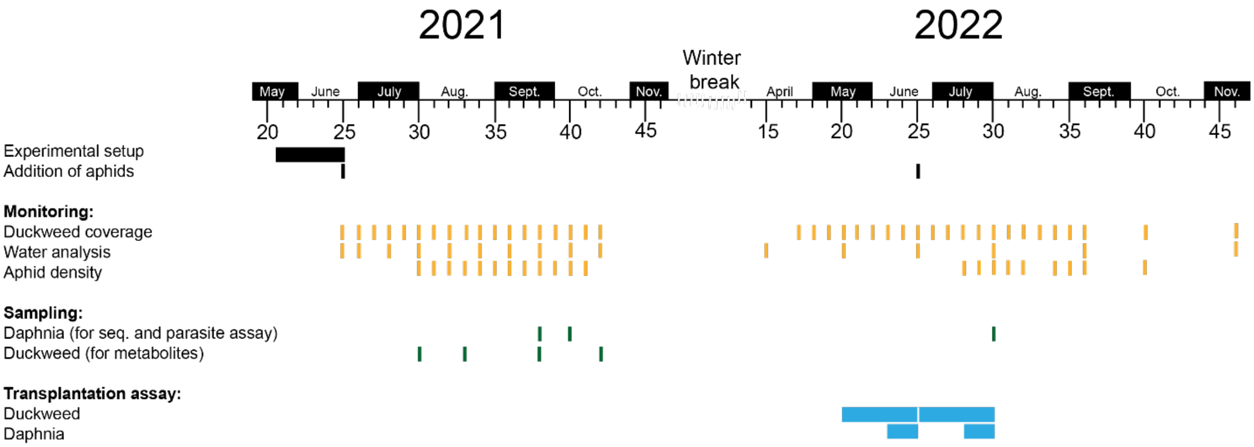

Fig. S1 Chronological outline of the experimental setup and sampling. Month and calendar week information is provided above and below the timeline, respectively.

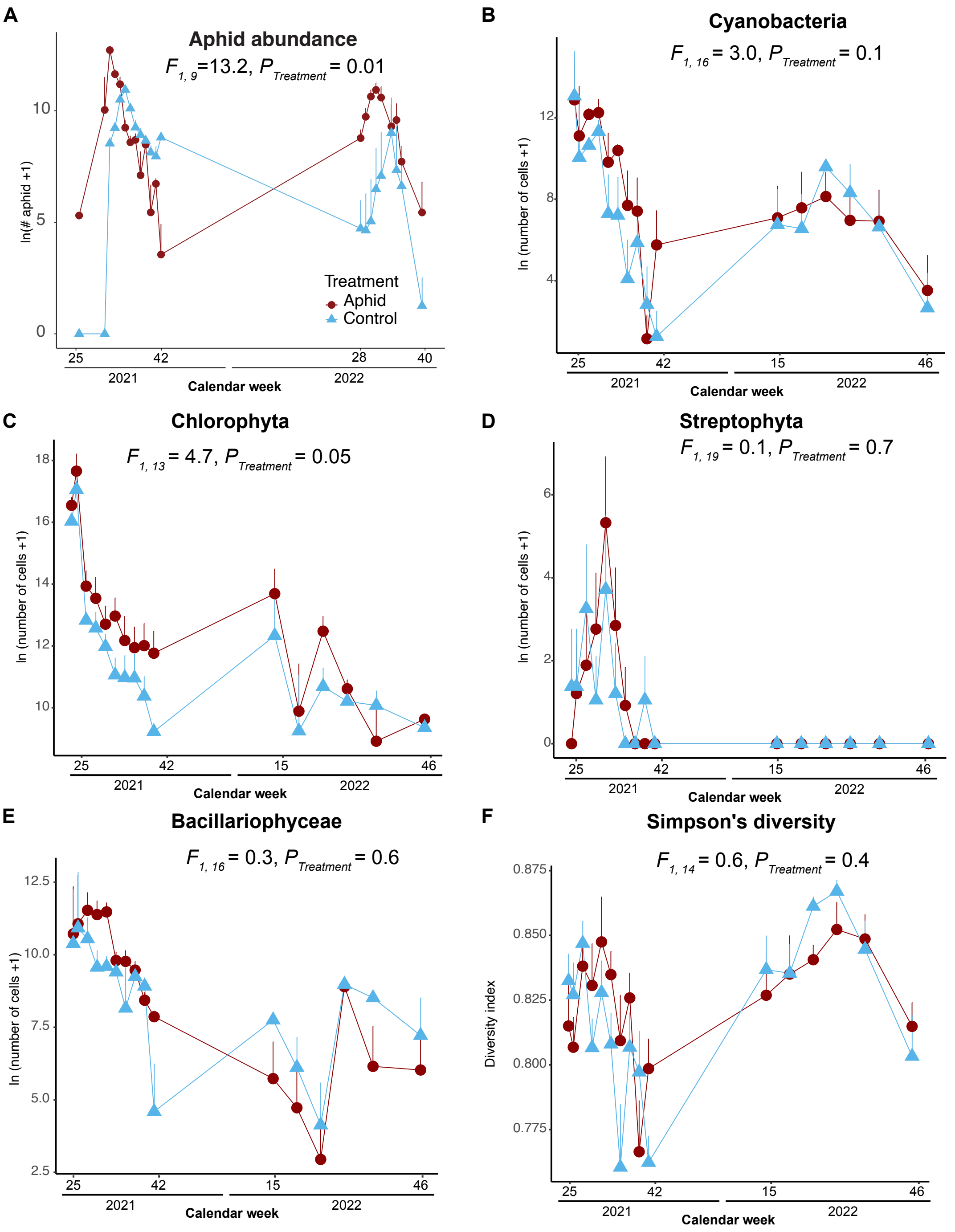

Fig. S2 Population changes among different species in the community. A-E refer to the abundance (per pond) of aphid (A), Cyanobacteria (B), Chlorophyta (C), Streptophyta (D), and Bacillariophyceae (E). Panel F refers to phytoplankton diversity in

the aquatic community, which is calculated using the log-transformed abundance (number of cells per liter) of each phytoplankton group. *P*-values refer to the effects of aphid herbivory. All *P*-values were estimated using mixed-effects models with time and pond ID as random factors. Light blue and red colors refer to control and aphid herbivory ponds. Error bars indicate to standard errors. Aphid abundance was estimated based on the aphid density (per frond) and duckweed coverage data.

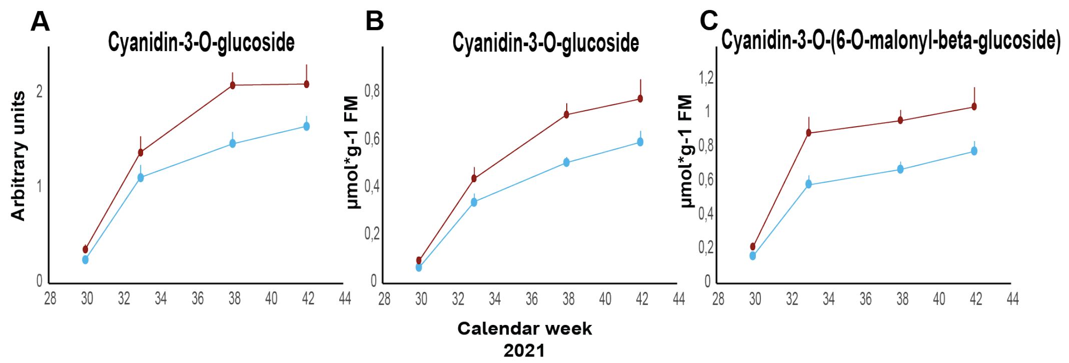

Fig. S3 Anthocyanin abundance in the duckweed populations.

The abundance of Cyanidin-3-O-glucoside (A, B) and Cyanidin-3-O-(6-O-malonyl-beta-glucoside) (C) in the duckweed plants at different time points in 2021. Light blue and red colors refer to control and aphid herbivory ponds. Data were either measured by LC-MS (A; relative quantification) or HPLC-PDA (B, C; absolute quantification). Error bars refer to standard errors.

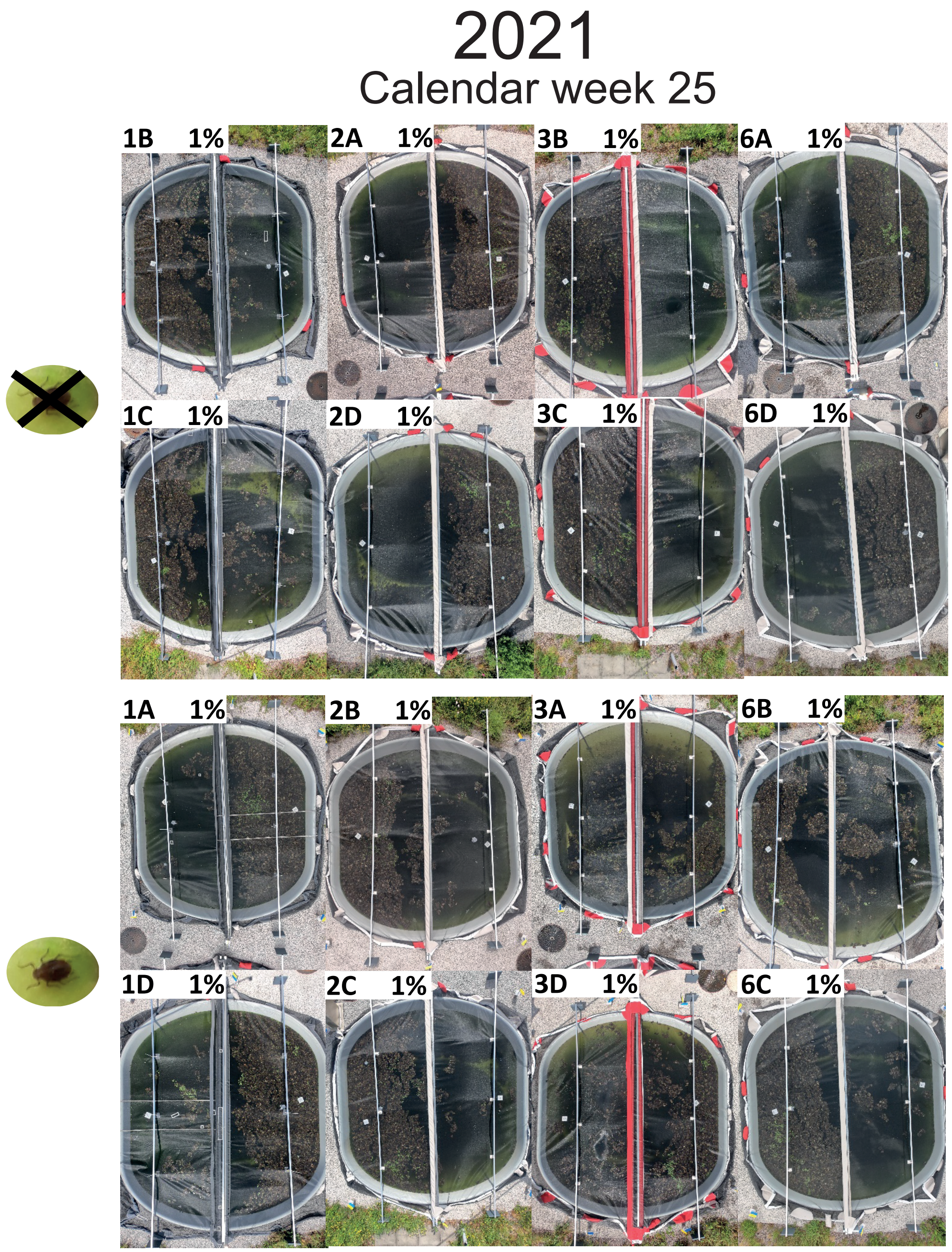

Fig. S4. Overview picture of the ponds at calendar week 25 in 2021. The pond ID and the estimated duckweed coverage levels are indicated in the upper left corner of each pond picture. In the upper part of the figure, the control ponds are shown, and in the lower part, the aphid herbivory ponds.

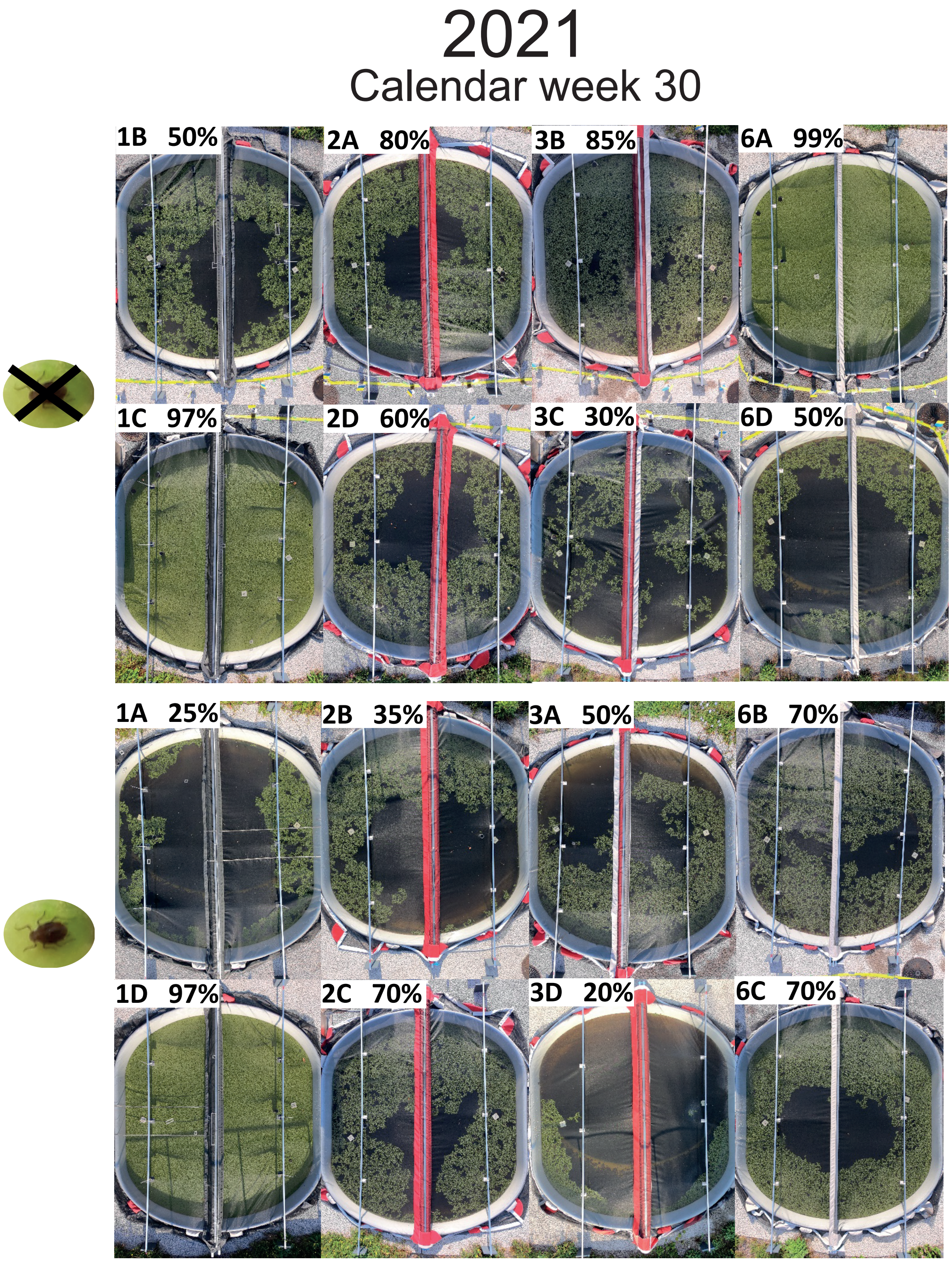

Fig. S5 Overview picture of the ponds at calendar week 30 in 2021. The pond ID and the estimated duckweed coverage levels are indicated in the upper left corner of each pond picture. In the upper part of the figure, the control ponds are shown, and in the lower part, the aphid herbivory ponds.

2021

Calendar week 32

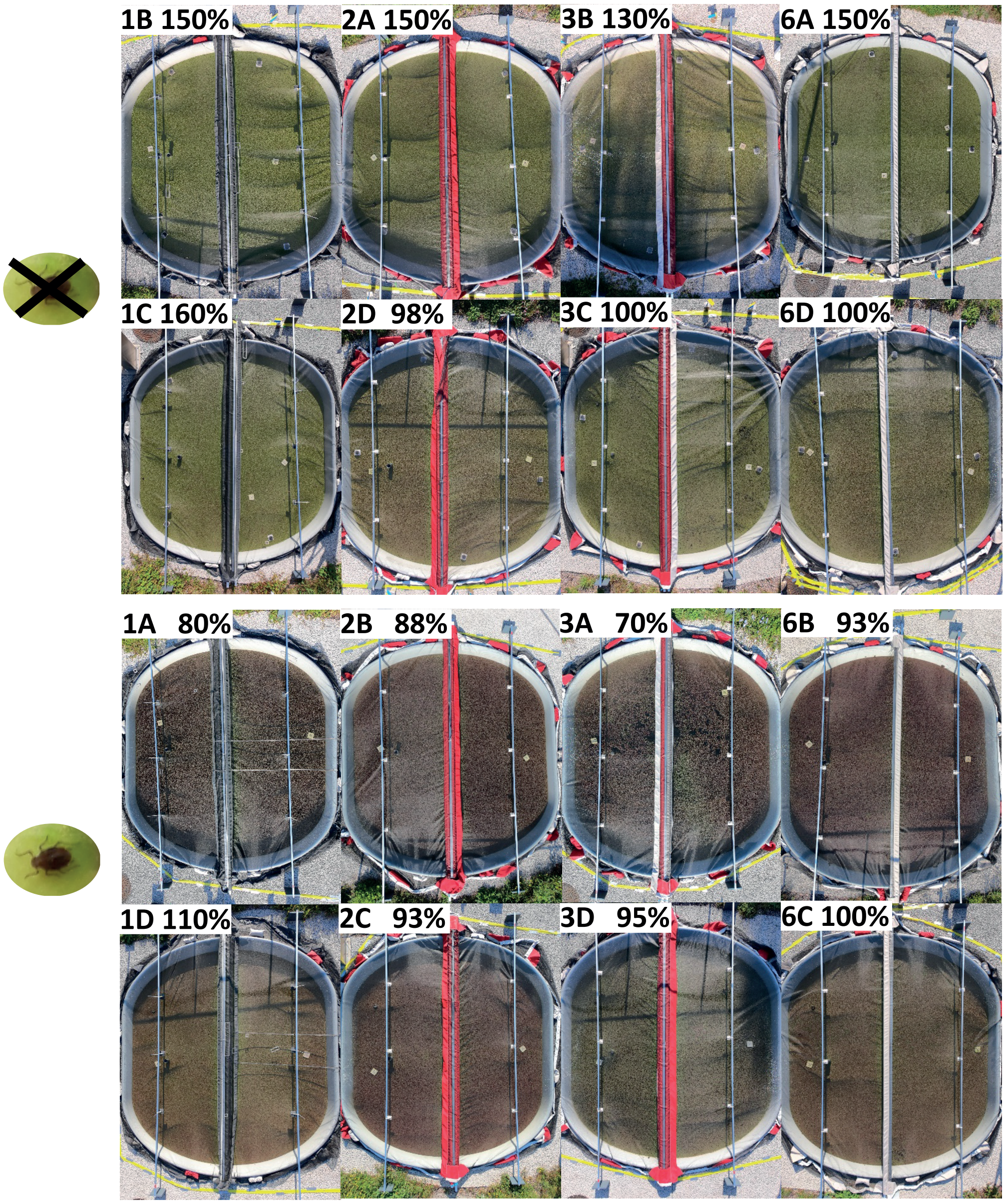

Fig. S6 Overview picture of the ponds at calendar week 32 in 2021. The pond ID and the estimated duckweed coverage levels are indicated in the upper left corner of each pond picture. In the upper part of the figure, the control ponds are shown, and in the lower part, the aphid herbivory ponds.

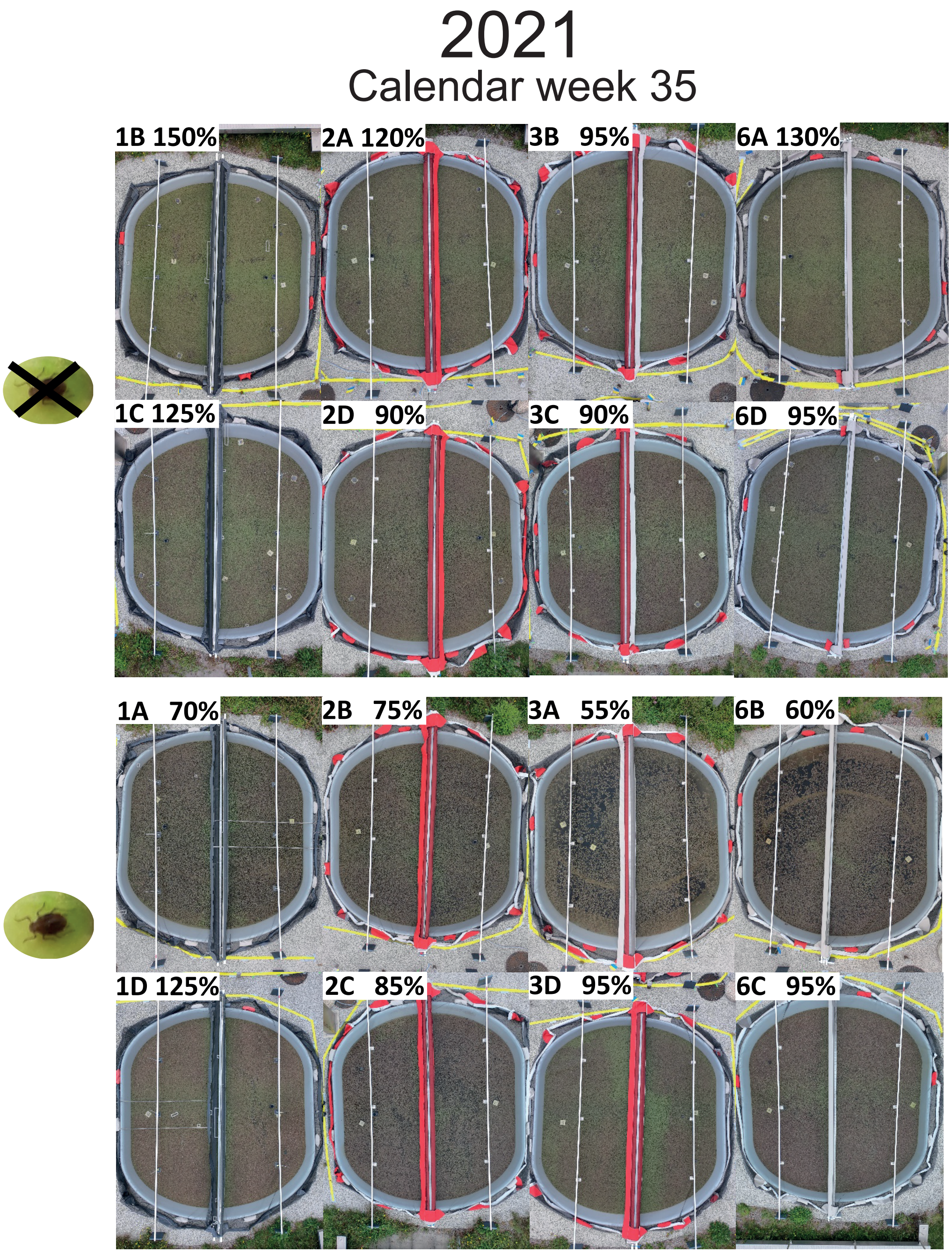

Fig. S7 Overview picture of the ponds at calendar week 35 in 2021. The pond ID and the estimated duckweed coverage levels are indicated in the upper left corner of each pond picture. In the upper part of the figure, the control ponds are shown, and in the lower part, the aphid herbivory ponds.

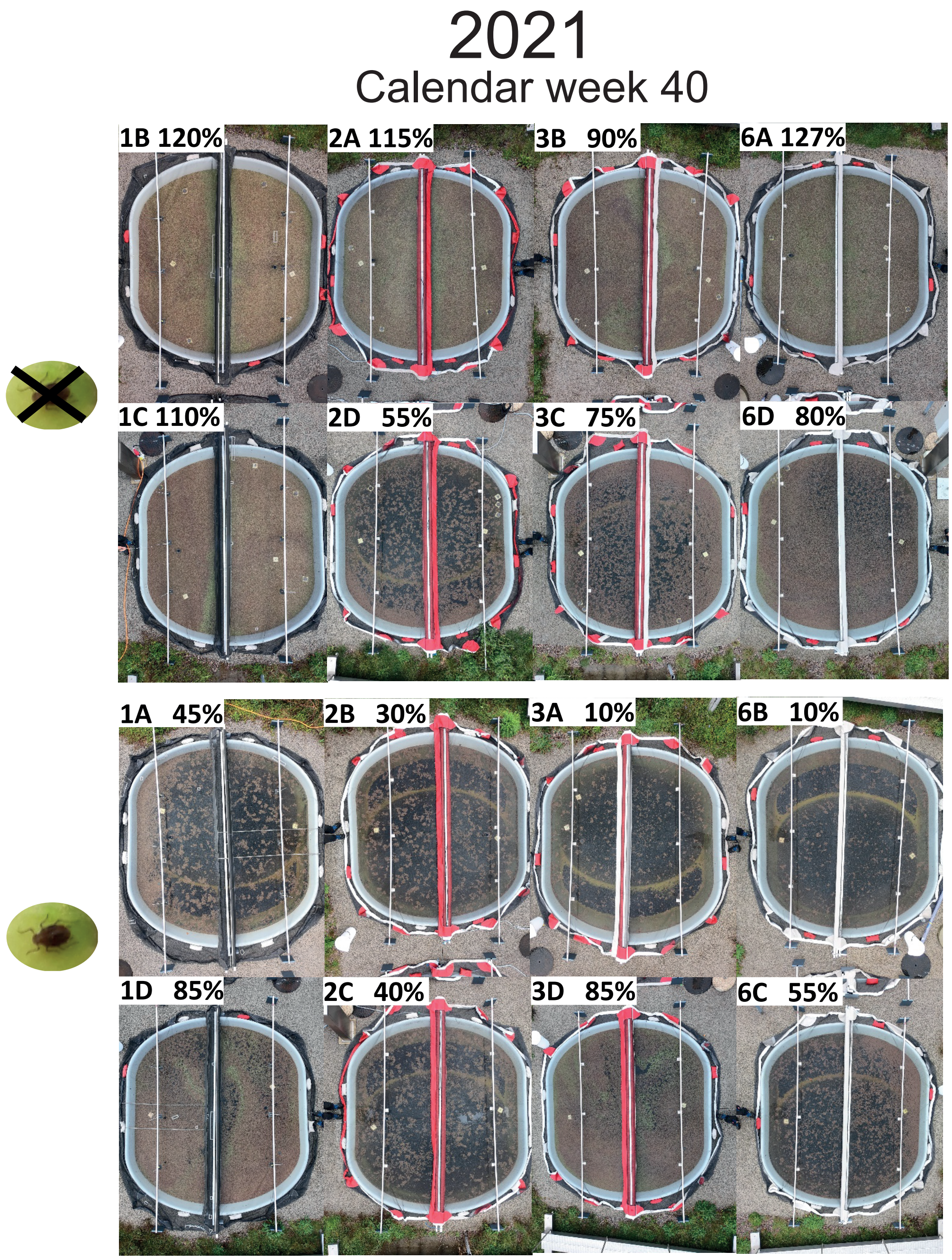

Fig. S8 Overview picture of the ponds at calendar week 40 in 2021. The pond ID and the estimated duckweed coverage levels are indicated in the upper left corner of each pond picture. In the upper part of the figure, the control ponds are shown, and in the lower part, the aphid herbivory ponds.

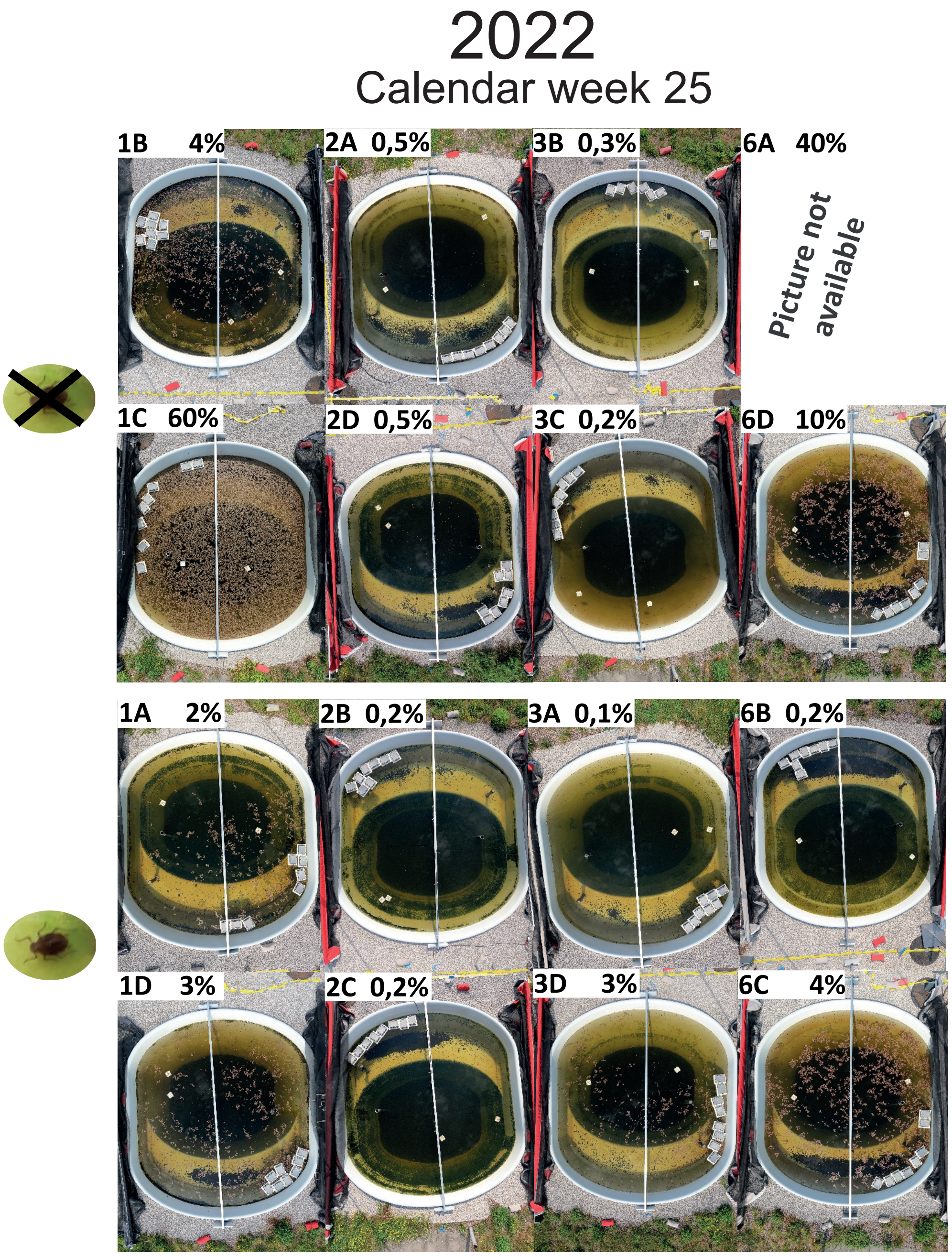

Fig. S9 Overview picture of the ponds at calendar week 25 in 2022. The pond ID and the estimated duckweed coverage levels are indicated in the upper left corner of each pond picture. In the upper part of the figure, the control ponds are shown, and in the lower part, the aphid herbivory ponds.

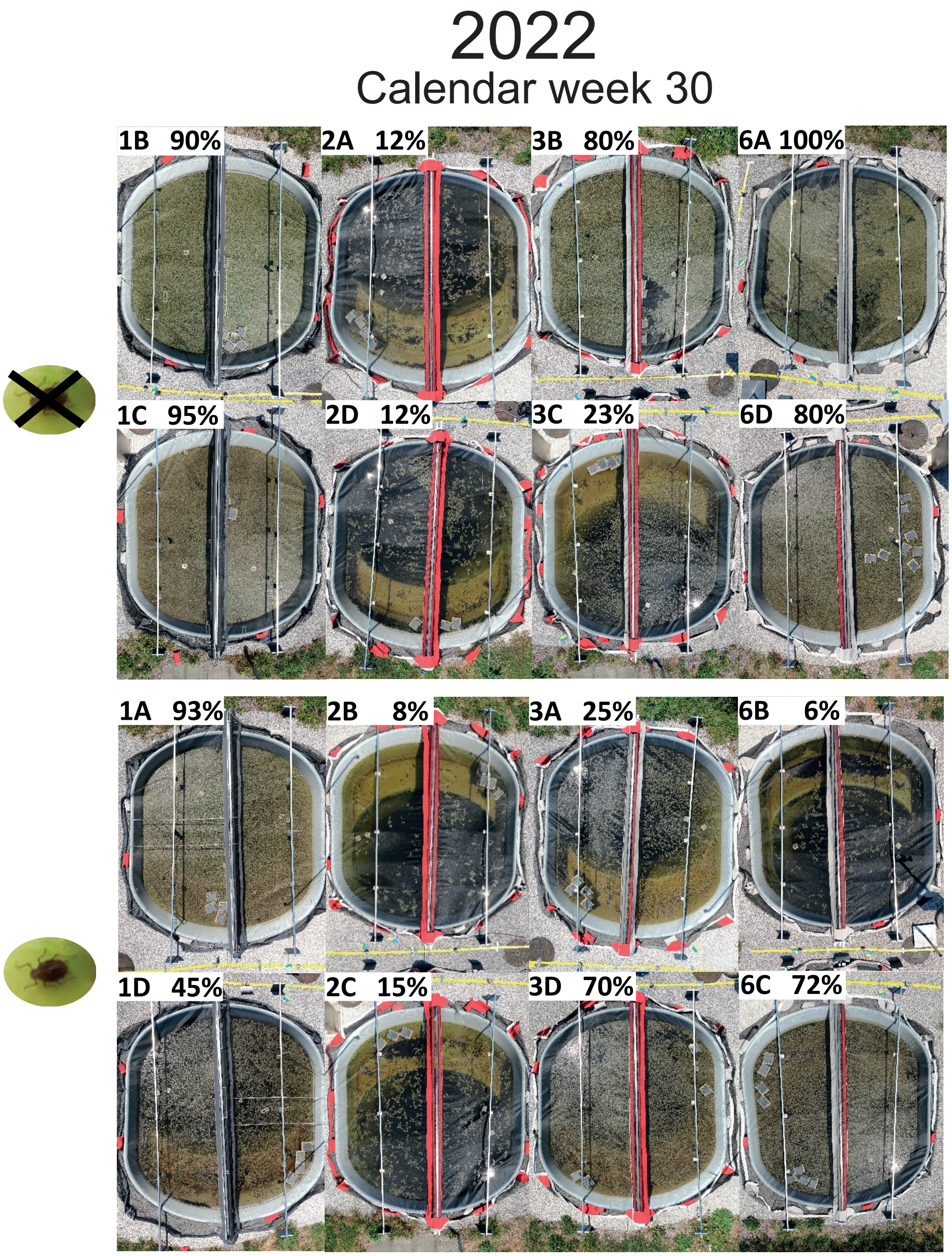

Fig. S10 Overview picture of the ponds at calendar week 30 in 2022. The pond ID and the estimated duckweed coverage levels are indicated in the upper left corner of each pond picture. In the upper part of the figure, the control ponds are shown, and in the lower part, the aphid herbivory ponds.

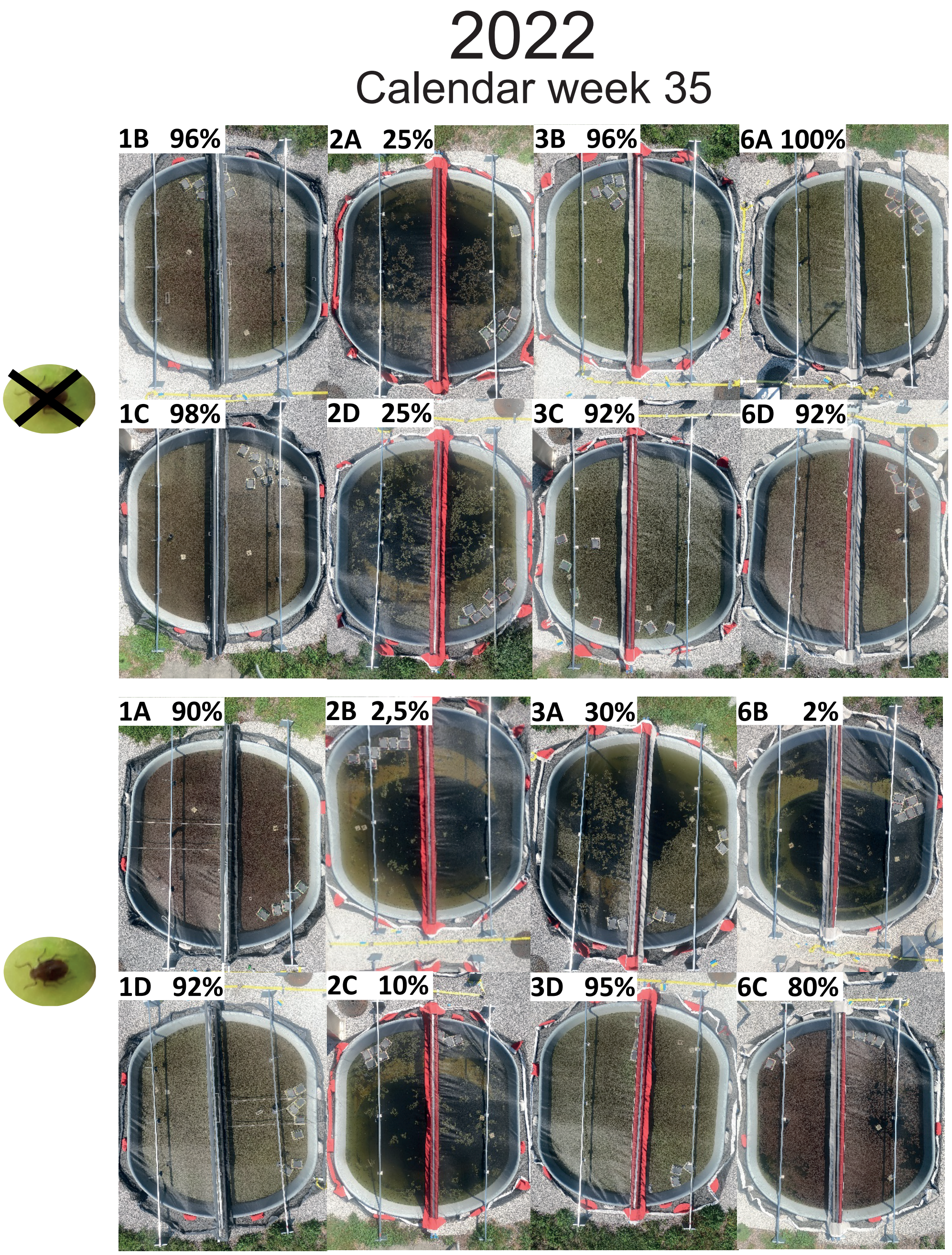

Fig. S11 Overview picture of the ponds at calendar week 35 in 2022. The pond ID and the estimated duckweed coverage levels are indicated in the upper left corner of each pond picture. In the upper part of the figure, the control ponds are shown, and in the lower part, the aphid herbivory ponds.

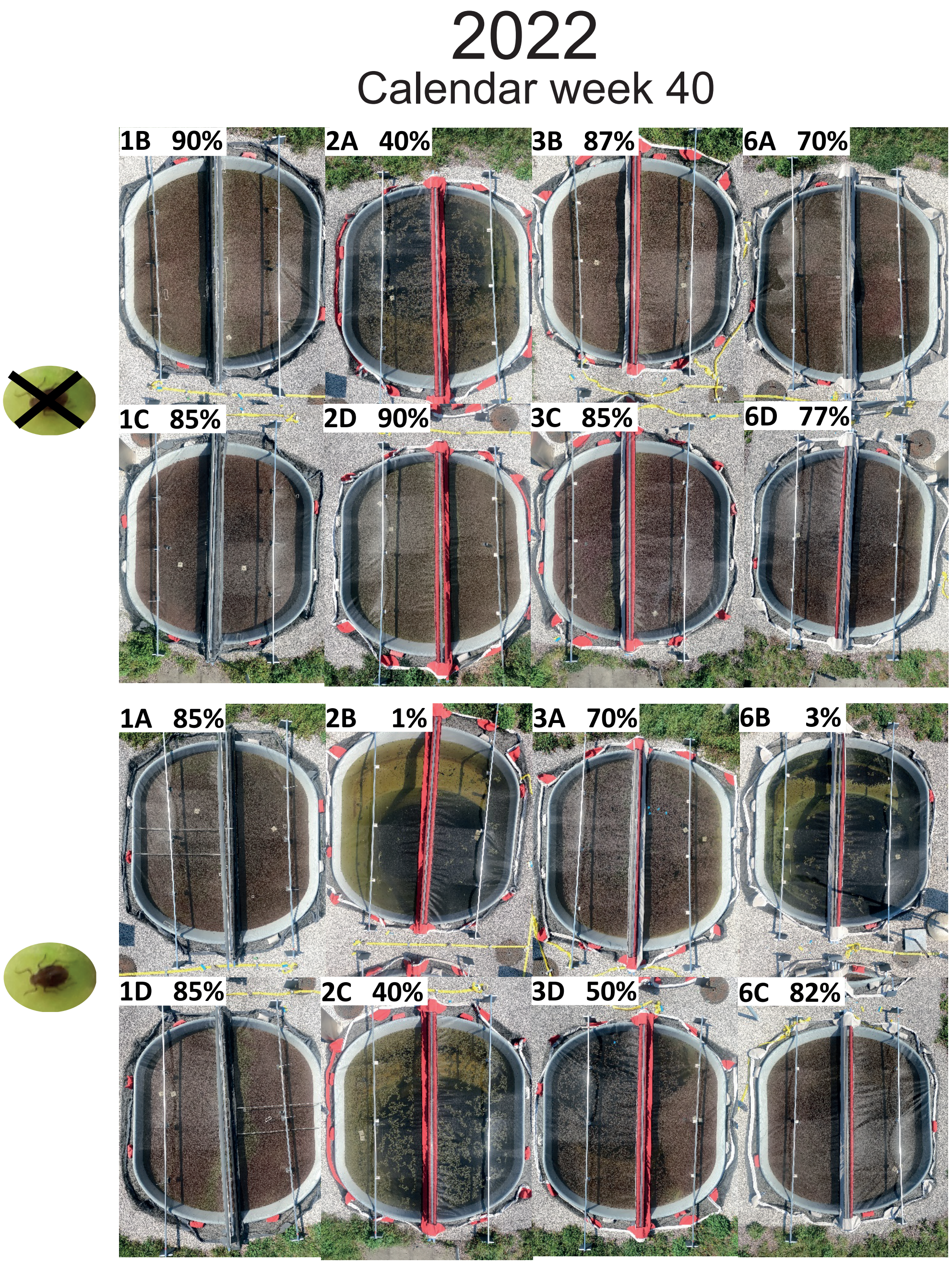

Fig. S12 Overview picture of the ponds at calendar week 40 in 2022. The pond ID and the estimated duckweed coverage levels are indicated in the upper left corner of each pond picture. In the upper part of the figure, the control ponds are shown, and in the lower part, the aphid herbivory ponds.

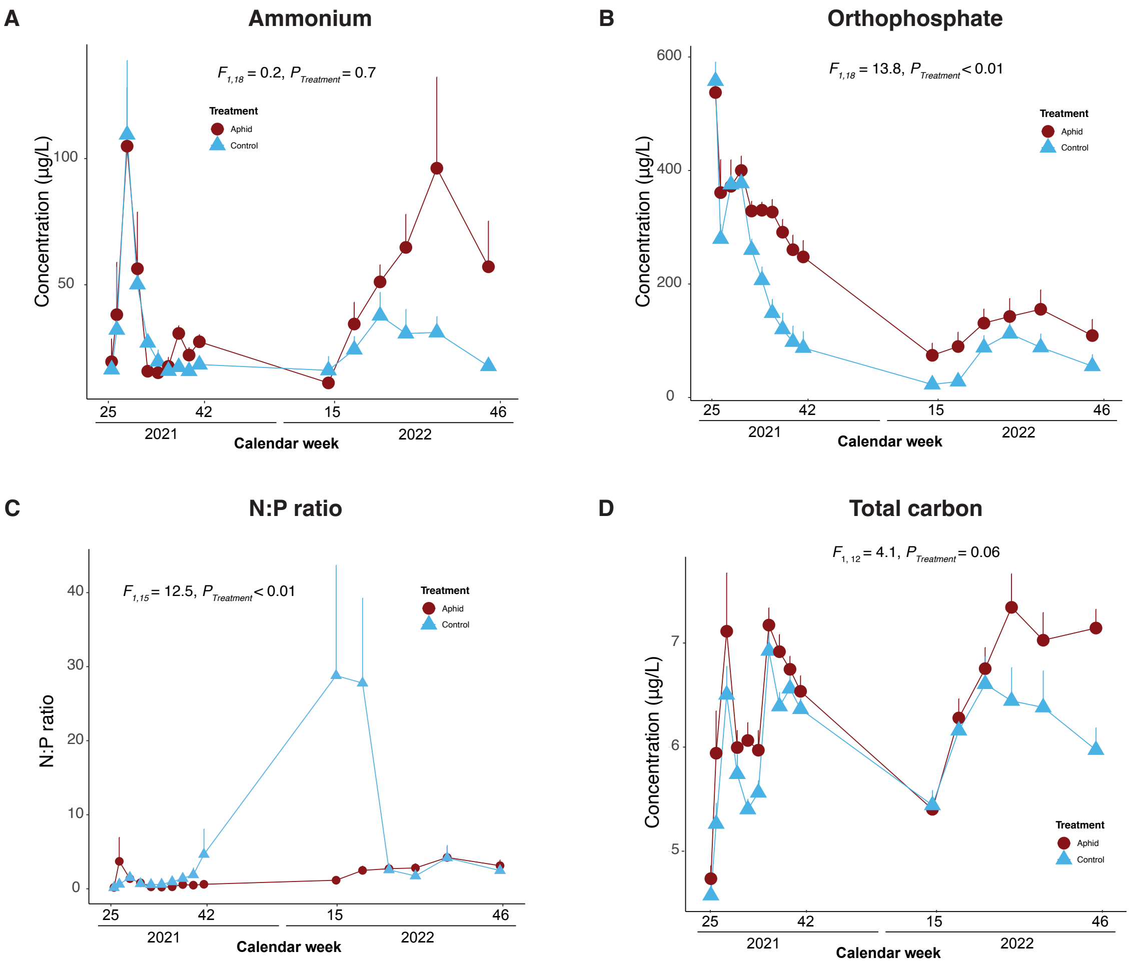

Fig. S13 Nutrient levels in the aquatic environment. A-D refer to the levels of ammonium nitrogen (A), orthophosphate (B), N:P molar ratio (C), and total carbon (D) in the water. *P*-values refer to the effects of aphid herbivory. All *P*-values were estimated using mixed-effects models with time and pond block as random factors. Light blue and red colors refer to control and aphid herbivory ponds. Error bars indicate to standard errors.

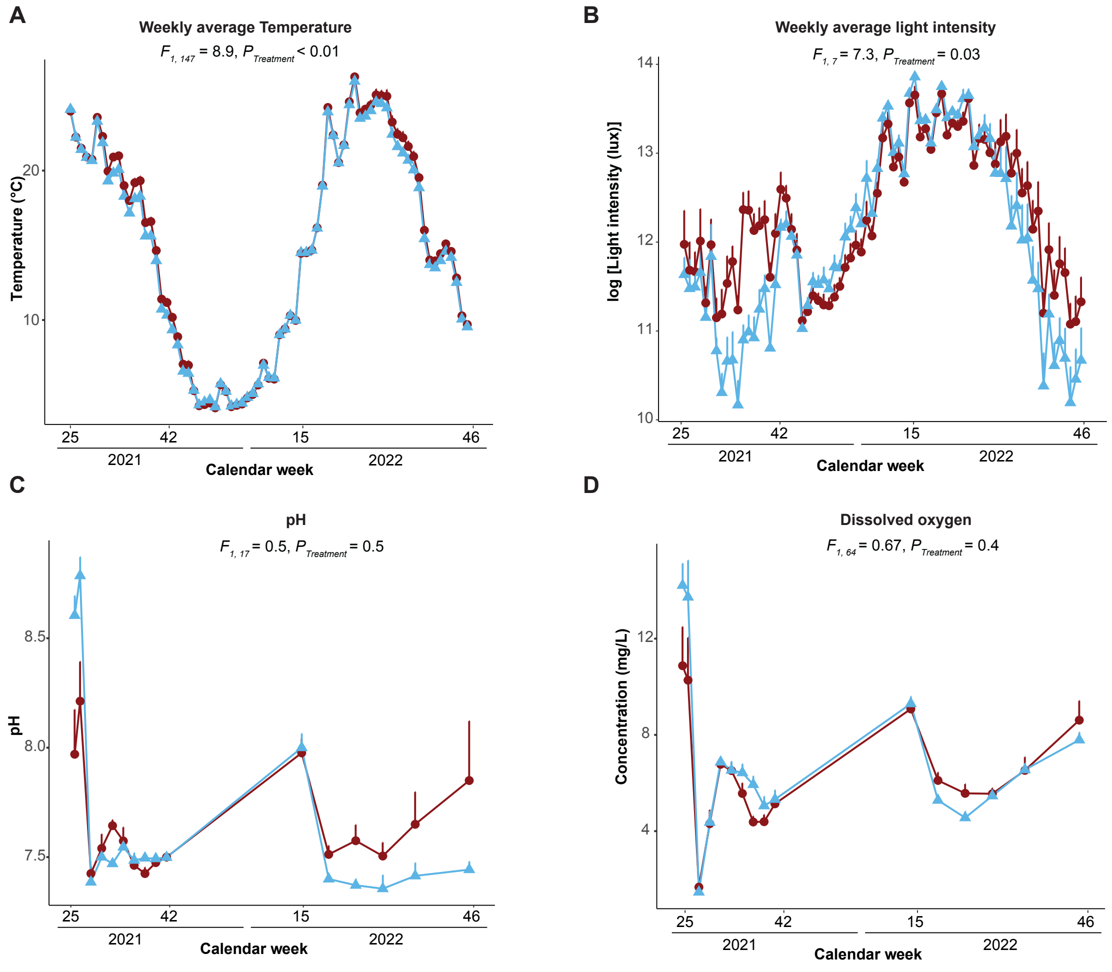

Fig. S14 Temperature, light intensity, pH, and oxygen levels in the ponds. A-D refer to the average weekly temperature (A), weekly average of the daily light intensity (B), pH (C), and dissolved oxygen levels (D). P-values refer to the effects of aphid herbivory. All P-values were estimated using mixed-effects models with time and pond block as random factors. Light blue and red colors refer to control and aphid herbivory ponds. Error bars refer to standard errors.

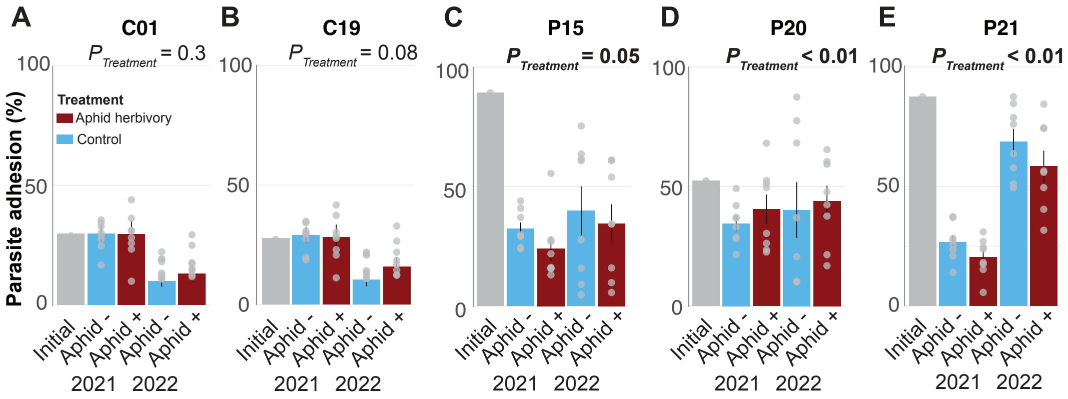

Fig. S15. Aphid-herbivory shifted *Daphnia magna* genotype frequencies. The genotype frequency changes in *D. magna* were quantified by measuring parasite adhesion phenotype to different parasite strains, which is determined by host genotype. Bar charts A-E illustrate changes in parasite adhesion phenotypes in *D. magna* populations for each of five strains of the parasite *P. ramosa*: C01, C19, P15, P20 and P21. *P*-values in A-E, determined using linear mixed-effects models with time and pond block as random factors, indicate the degree to which aphid herbivory affected attachment in *D. magna* populations for each parasite strain. Grey bars refer to the frequencies of adhesion phenotype in the initial population. Blue and dark red bars refer to control and aphid-herbivory treatments, respectively. Error bars depict one standard error. In each panel, the individual adhesion level from each population is shown as light gray dots.

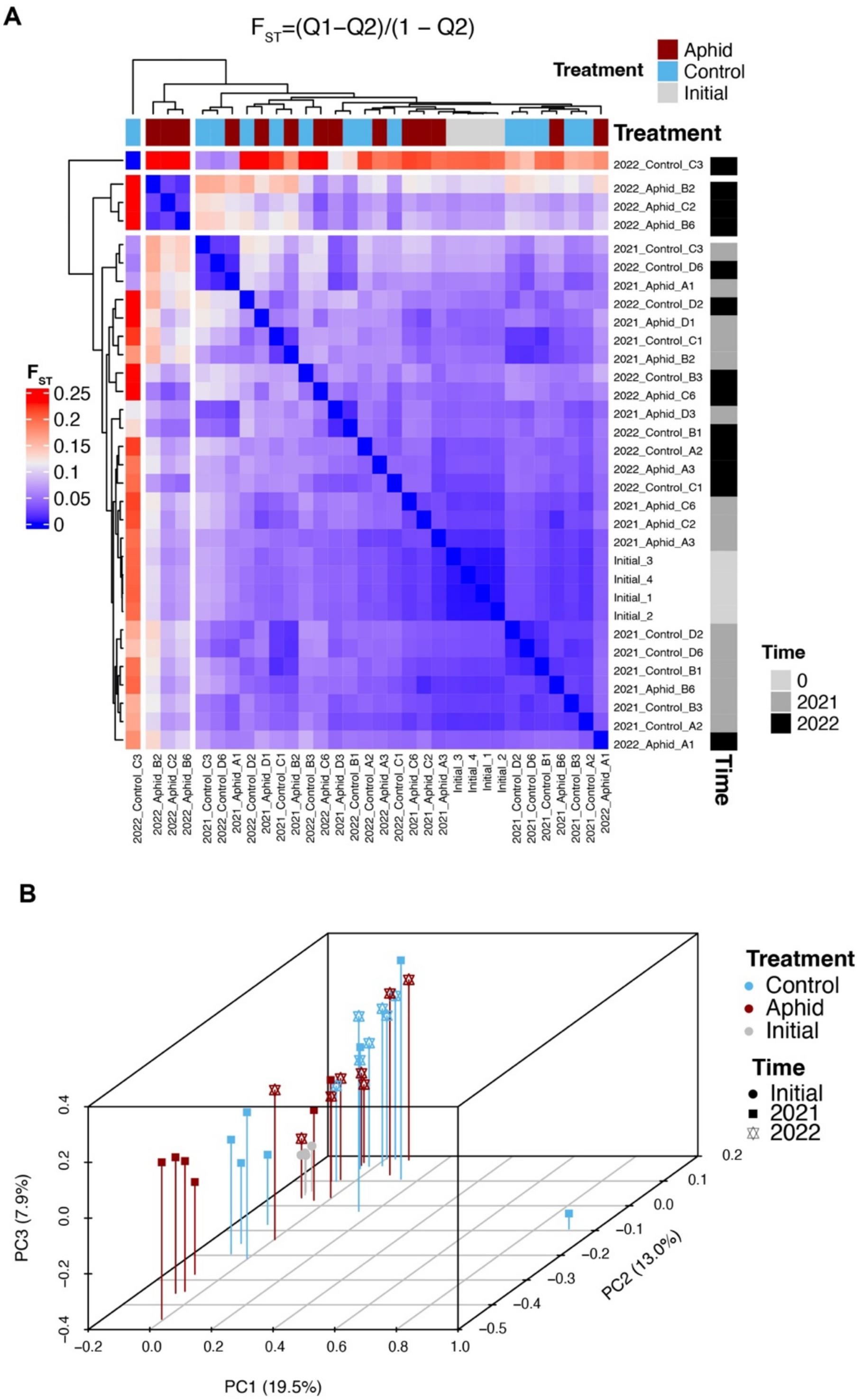

Fig. S16 Genomic differentiation among populations. A: heatmap shows the similarity among *D. magna* populations based on pairwise F_{st}. B: principal component

plot shows the variations among evolved and initial *D. magna* populations. Among all samples, pond 3C in 2022 showed the most difference from the others. The four initial populations showed the lowest F_{ST} among each other. Populations from 2022 showed increased F_{ST} to other populations.

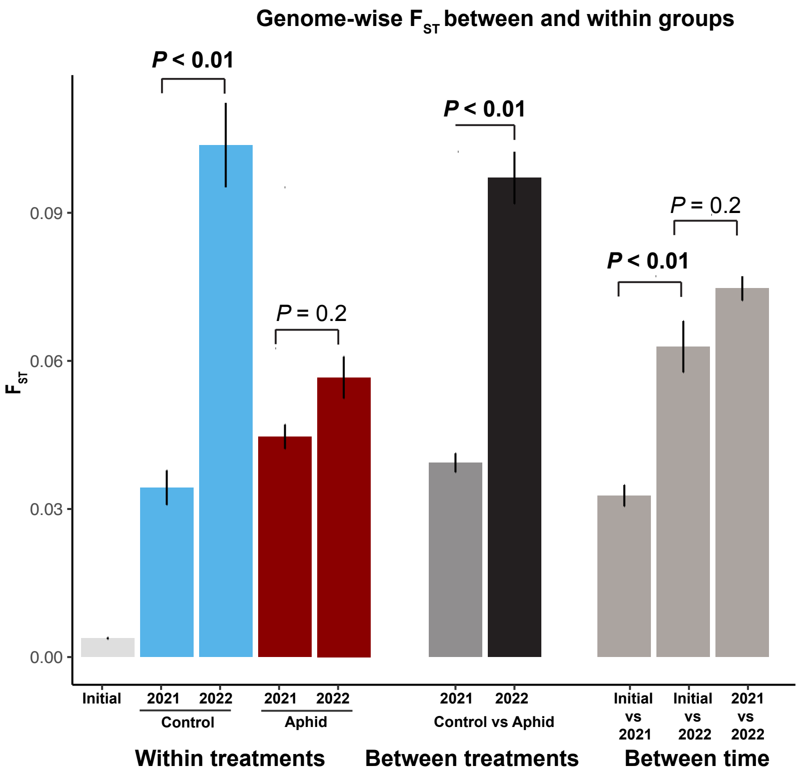

Fig. S17 Genomic differentiation of *D. magna* within and between treatments and time. The y-axis refers to the Fst values. The x-axis refers to different comparisons. Mean and standard errors are shown. P-values were estimated using one-way ANOVA.

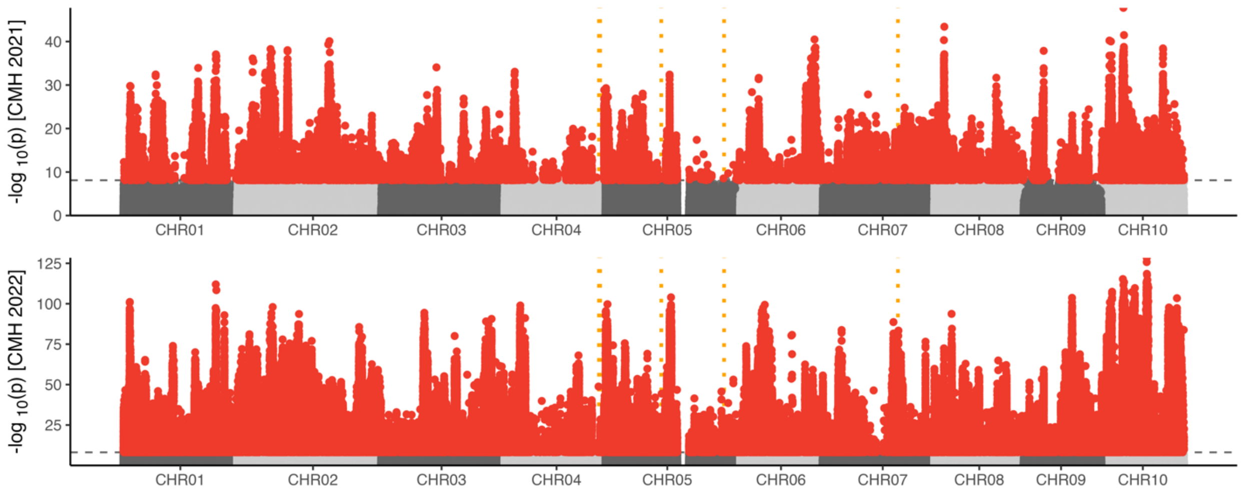

Fig. S18 Cochran–Mantel–Haenszel test showing *D. magna* genomic divergence between control and aphid-herbivory populations. The y-axis refers to $-\log_{10}$ P-values. The x-axis refers to the genomic position of each SNP. The horizontal dashed lines show the $P < 0.05$ cutoffs after the Bonferroni correction. Significant SNPs are shown in red. The vertical lines in orange refer to the location of previously identified loci that affect *Pasteuria ramosa* attachment ability to *D. magna*. Samples collected in 2021 and 2022 are shown in upper and lower panels, respectively.

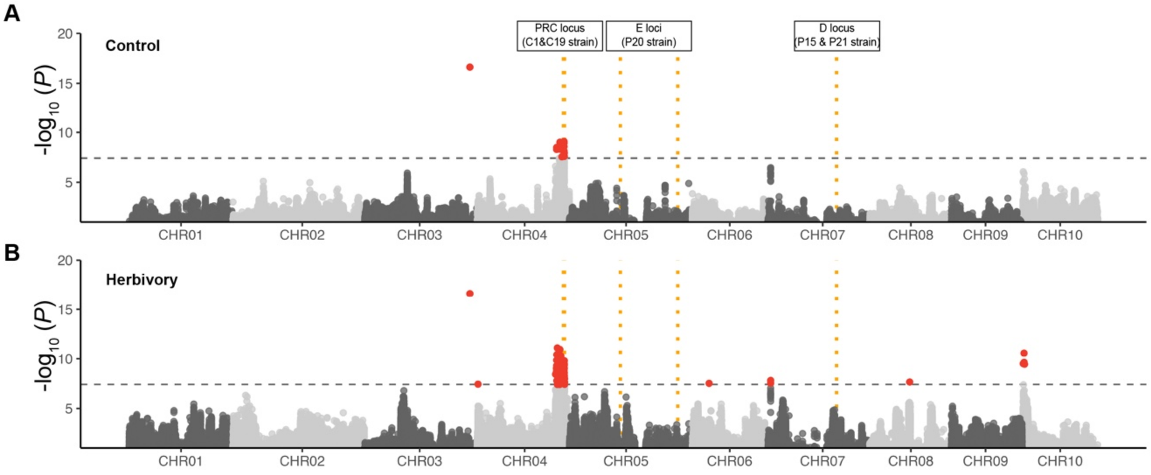

Fig. S19 Genomic selection on the *D. magna* populations. The y-axis refers to -$\log_{10}$ P-values. X-axis refers to the genomic position of each SNP. The populations evolved in control (A) and aphid herbivory ponds (B) are shown in the upper and lower panels, respectively. The horizontal dashed lines show the $P < 0.05$ cutoff after the Bonferroni correction. Significant SNPs are shown in red. The vertical lines in orange refer to the location of previously identified loci that affect *Pasteuria ramosa* attachment ability to *D. magna*. PRC: *Pasteuria* resistance complex.

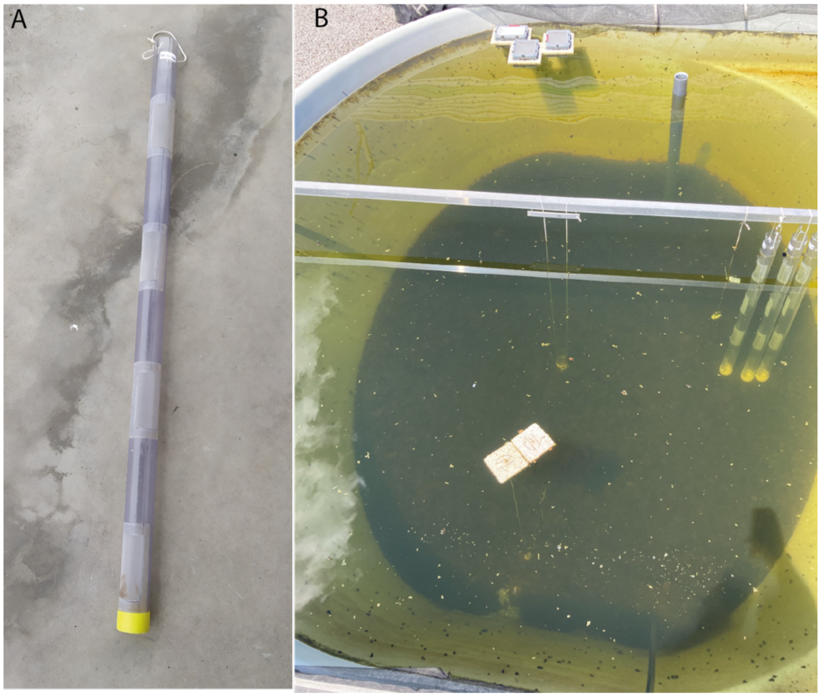

Fig. S20 Setup of the *Daphnia magna* transplant experiments. (A) For the *D. magna* transplant experiment, we used PVC columns that contained various cut-outs covered by mesh to allow for an exchange of water and phytoplankton while retaining *D. magna*. (B) PVC columns containing the *Daphnia* were placed in the ponds next to each other by attaching them to the metal bar in the middle of the ponds.

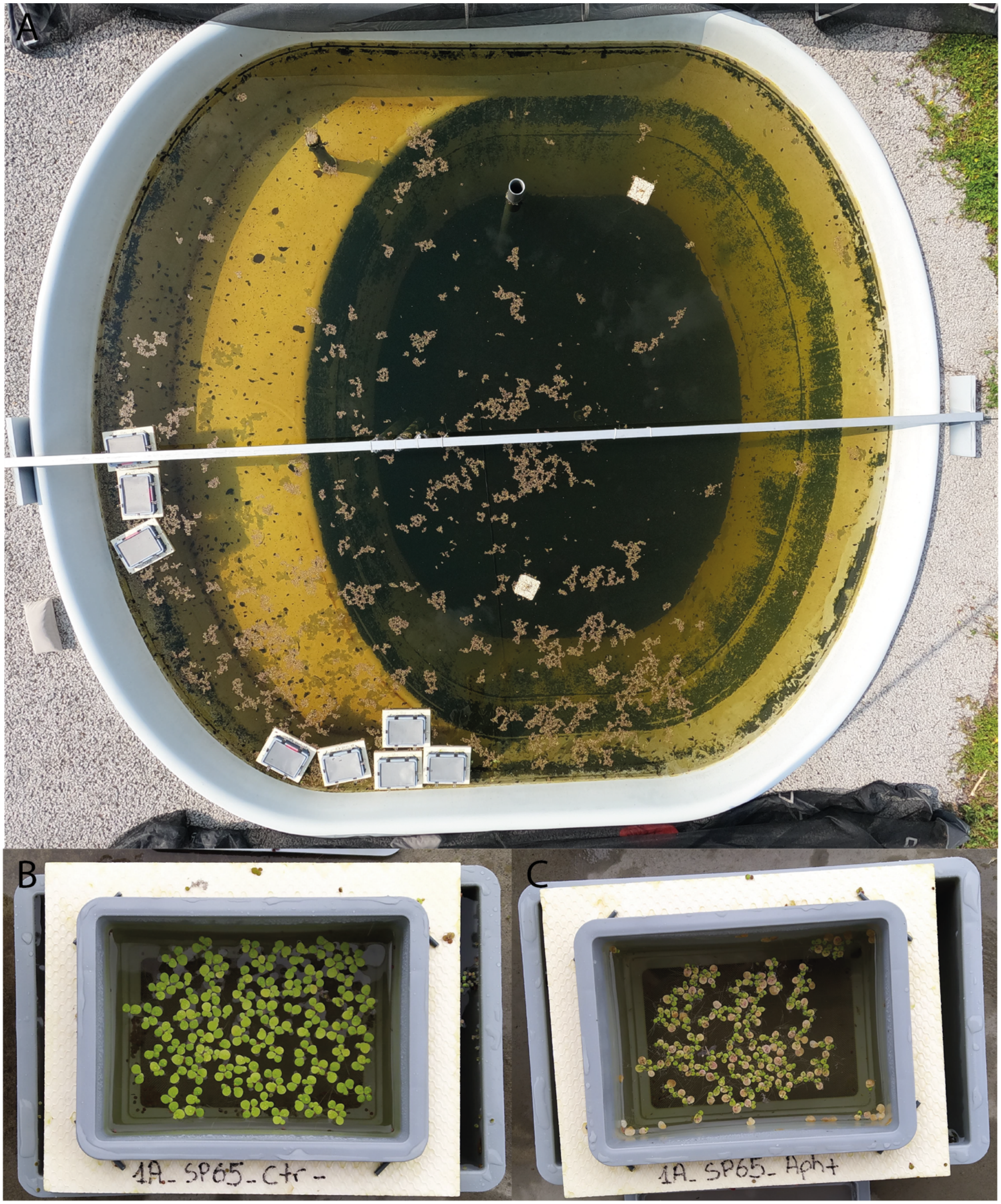

Fig. S21 Setup of the *Spirodela polyrhiza* and aphid transplant assays within the ponds. (A) Swimming boxes containing the duckweed of the transplant experiment were allowed to move freely on the water. Exemplary swimming boxes with control (B) and

aphid herbivory treatment (C). During sampling and analysis, boxes were placed in a slightly bigger, closed box filled with pond water to temporarily take them out of the ponds (B, C).

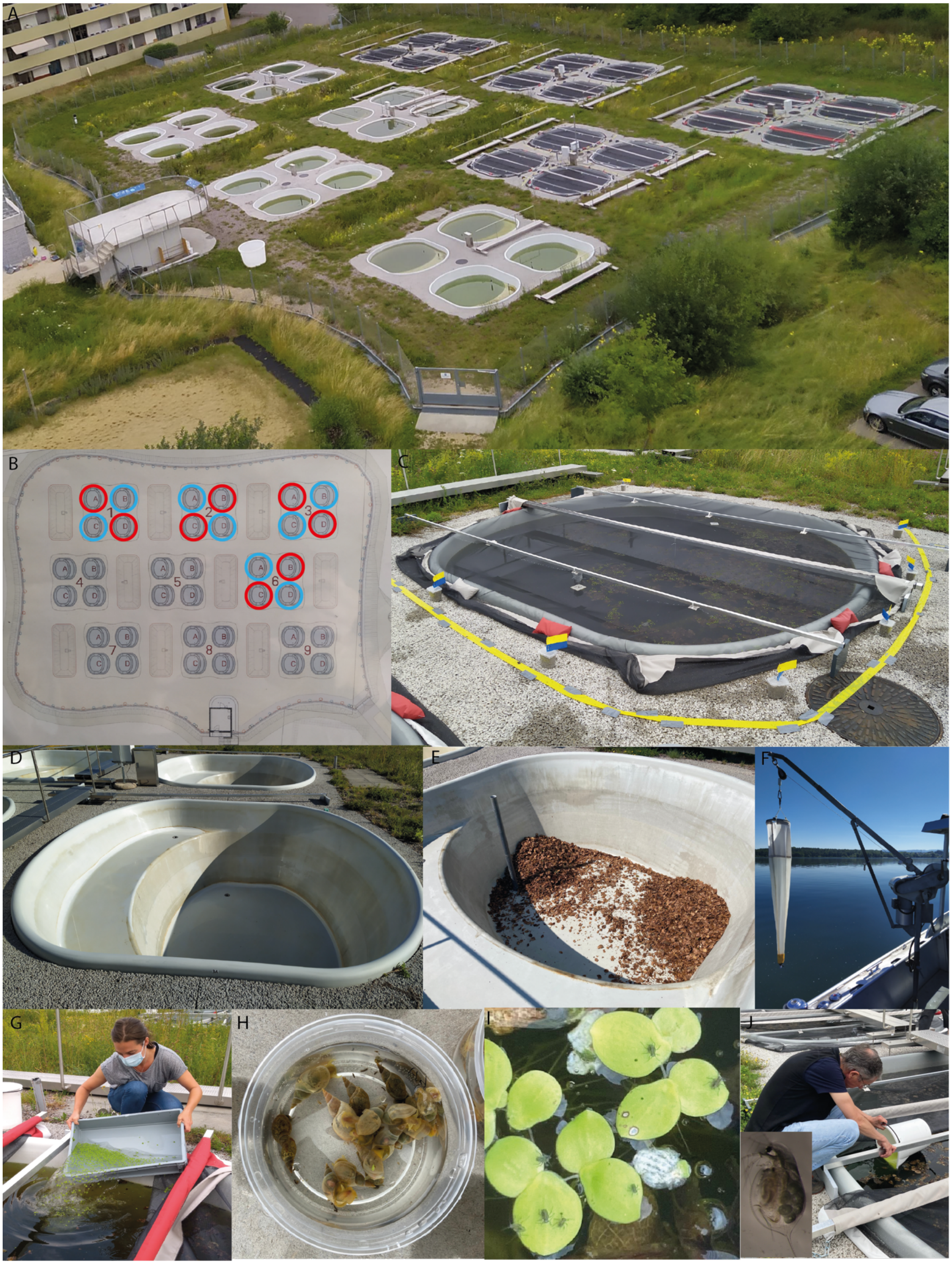
A
B
C
D
E
F
G
H
I
J

Fig. S22 Experimental setup. (A) Overview of the pond facility at Eawag. (B) Scheme of the used ponds and their designation. Ponds used for the experiment are highlighted. Light blue and red colors refer to control and aphid herbivory ponds. (C) Close-up of a fully prepared pond with mesh cover and the aphid protection measures (yellow and blue sticky traps) around. (D) Empty pond before. (E) Pond with leaf litter. (F) Equipment that was used to sample the plankton within water columns at Greifensee for the inoculation of the ponds at the beginning of the experiment. Addition of (G) duckweed, (H) snails, (I) aphids, and (J) daphnia at the start of the experiment.

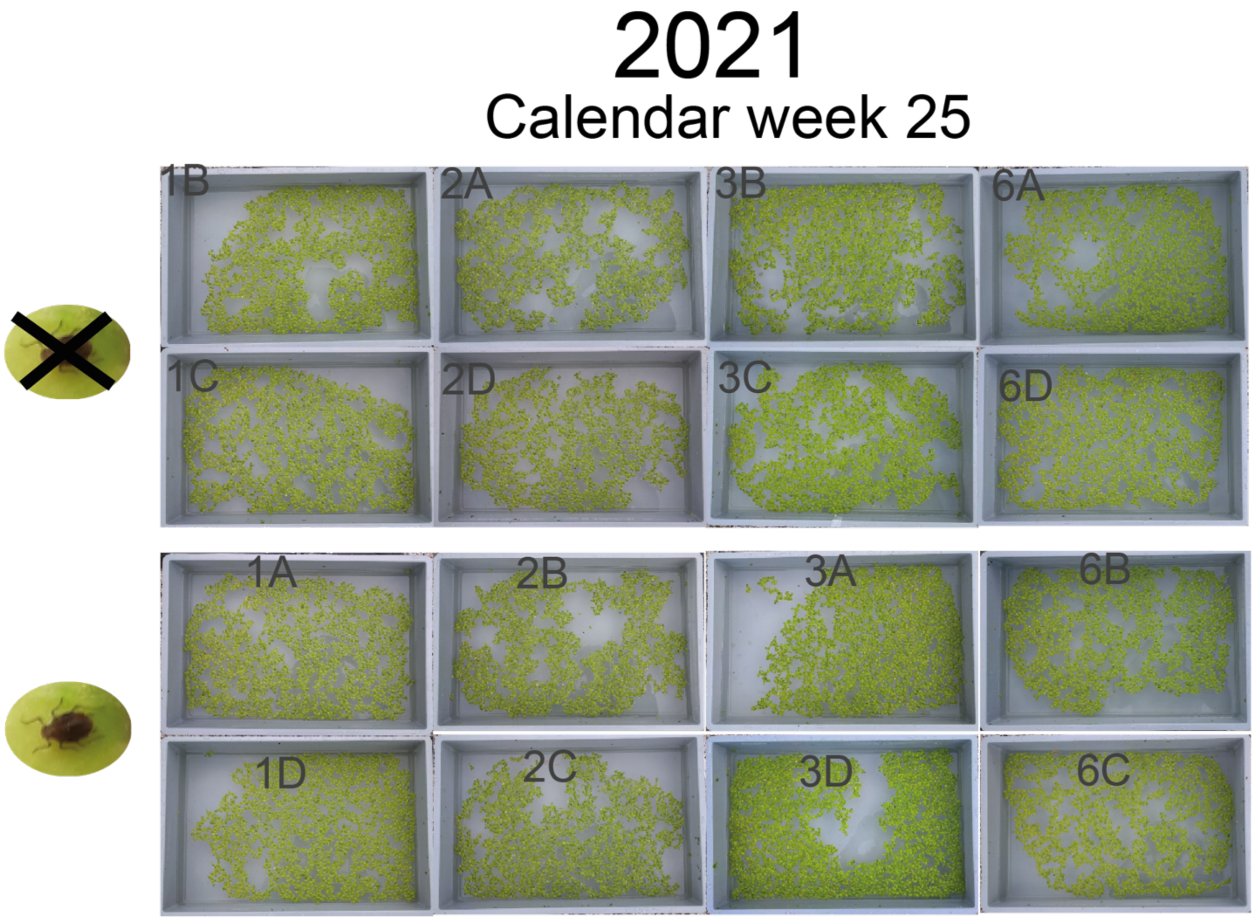

Fig. S23 Overview picture of the duckweed used for the pond inoculation at the start of the experiment. *Spirodela polyrhiza* fronds before addition to the ponds in calendar week 25 in 2021. The pond ID, which the material corresponds to, is indicated in gray. In the upper part, the material for the control ponds is shown, and in the lower part, those of the aphid herbivory ponds. Pond 6A was removed from the analysis in 2021.

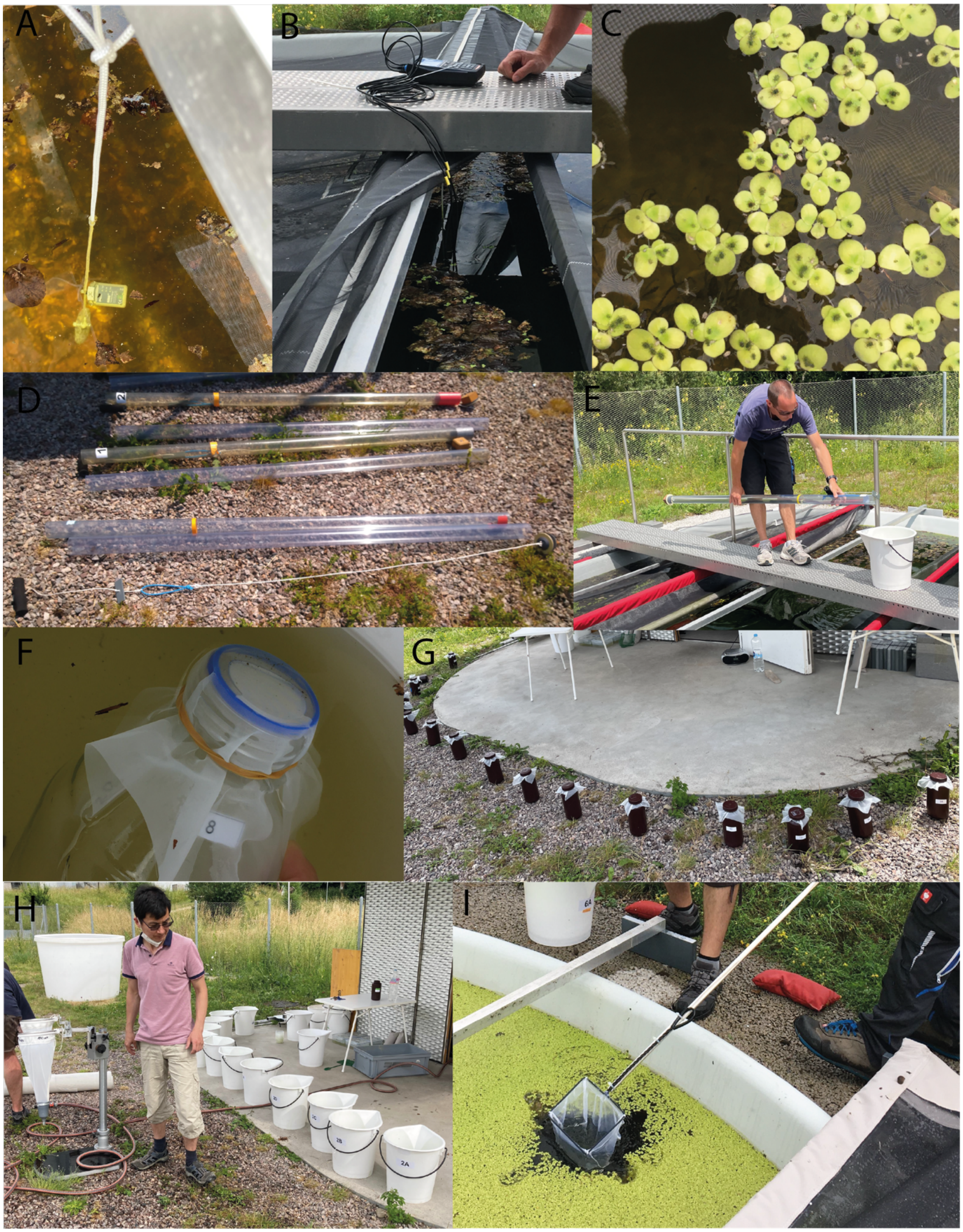

Fig. S24 Data collection and sampling procedure (A) Data loggers for the continuous recording of temperature and light data. (B) Dipping sonde for measuring the oxygen, pH

and conductivity. (C) An exemplary picture was used to estimate aphid densities. (D, E) Leibold-samplers that were used for the collection of water columns. Subsampling of the water used for (F) nutrient analysis, as well as (G) chlorophyll and phytoplankton analysis. (H) Buckets with the collected water and, on the left, the filtering equipment (mesh funnels) to collect the zooplankton. (I) Collection of *D. magna*.

Table S1. Summary of the statistical results on the effects of aphid herbivory on aquatic communities. The results were obtained from linear mixed-effects models with this formula: lmer(Response~AphidTreatment*Year + poly(Week, 2) + (1|PondID), REML = T). The effect size (partial η^2) is calculated by the formula: Effect size = (F × df_effect) / (F × df_effect + df_error). Since the aphid effects can only be seen a few weeks after we have introduced the aphids, for phytoplankton responses, we excluded the first 2 weeks' data points from the statistical analysis. For D. magna and nutrient analysis, we excluded the data from the first 7 weeks, as the responses are expected to be seen later.

Response variable	Explanatory variable	Numerator df	Denominator df	*F* value	*P* value	Effect size
Aphid density	Treatment	1	12	12.1	**4.4e-03**	0.07
	Year	1	15	0.6	4.6E-01	0
	poly(Week, 2)	2	70	18.3	3.9E-07	0.2
	Treatment:Year	1	12	16.2	**1.5E-03**	0.1
Duckweed coverage	Treatment	1	13	14.0	**2.5E-03**	0.08
	Year	1	10	19.7	1.3E-03	0.1
	poly(Week, 2)	2	118	44.8	3.4E-15	0.4
	Treatment:Year	1	14	7.2	**1.8E-02**	0.04
ChlA	Treatment	1	73	3.8	5.6E-02	0.02
	Year	1	21	1.4	2.5E-01	0.01
	poly(Week, 2)	2	196	0.1	8.9E-01	0
	Treatment:Year	1	19	4.2	5.6E-02	0.02
D. magna	Treatment	1	13	6.4	**2.5E-02**	0.04
	Year	1	15	11.3	4.4E-03	0.06
	poly(Week, 2)	2	132	23.2	2.3E-09	0.2
	Treatment:Year	1	18	2.1	1.6E-01	0.01
Ammonium	Treatment	1	18	0.2	6.6E-01	0
	Year	1	10	11.6	6.3E-03	0.06
	poly(Week, 2)	2	78	16.8	8.4E-07	0.2
	Treatment:Year	1	28	0.3	5.9E-01	0
Phosphate	Treatment	1	14	13.8	**2.2E-03**	0.08
	Year	1	15	73.5	4.2E-07	0.3

	poly(Week, 2)	2	128	28.9	4.5E-11	0.3
	Treatment:Year	1	21	35.8	**6.5E-06**	0.2
N:P ratio	Treatment	1	14	9.5	**8.2E-03**	0.05
	Year	1	6	0.06	8.2E-01	0
	poly(Week, 2)	2	96	9.3	1.9E-04	0.1
	Treatment:Year	1	8	7.4	**2.4E-02**	0.04
Total carbon	Treatment	1	12	4.1	6.5E-02	0.02
	Year	1	12	3.0	1.1E-01	0.02
	poly(Week, 2)	2	99	28.3	1.9E-10	0.3
	Treatment:Year	1	24	0.6	4.4E-01	0
Temperature	Treatment	1	147	8.9	**3.4E-03**	0.05
	Year	1	147	270.4	4E-35	0.6
	poly(Week, 2)	2	147	916.3	1E-83	0.9
	Treatment:Year	1	147	0.8	3.7E-01	0
O_2	Treatment	1	64	0.7	4.2E-01	0
	Year	1	12	2.2	1.6E-01	0.01
	poly(Week, 2)	2	139	29.7	1.8E-11	0.3
	Treatment:Year	1	16	3.9	6.7E-02	0.02
pH	Treatment	1	17	0.5	4.9E-01	0
	Year	1	13	1.2	3E-01	0.01
	poly(Week, 2)	2	112	33.2	4.6E-12	0.3
	Treatment:Year	1	24	0.9	3.6E-01	0.01
Light intensity (lux)	Treatment	1	7	7.3	**2.9E-02**	0.04
	Year	1	53	17.8	9.6E-05	0.09
	poly(Week, 2)	2	86	34.0	1.3E-11	0.3
	Treatment:Year	1	54	9.3	**3.5E-03**	0.05
Chlorophyta	Treatment	1	11	4.7	**5.3E-02**	0.03
	Year	1	19	29.2	3.2E-05	0.2
	poly(Week, 2)	2	185	16.9	1.8E-07	0.2
	Treatment:Year	1	18	1.6	2.3E-01	0.01
Bacillariophyceae	Treatment	1	16	0.3	6.2E-01	0
	Year	1	25	29.6	1.2E-05	0.2
	poly(Week, 2)	2	192	4.9	8.5E-03	0.05
	Treatment:Year	1	25	11.0	2.8E-03	0.06
Streptophyta	Treatment	1	19	0.1	7.3E-01	0
	Year	1	17	6.0	2.5E-02	0.03
	poly(Week, 2)	2	176	5.6	4.5E-03	0.06
	Treatment:Year	1	26	0.1	7.6E-01	0
Cyanobacteria	Treatment	1	16	3.0	1E-01	0.02
	Year	1	31	0.4	5.2E-01	0
	poly(Week, 2)	2	191	34.8	1.3E-13	0.3
	Treatment:Year	1	28	1.9	1.8E-01	0.01

Simpson's diversity	Treatment	1	14	0.6	4.4E-01	0
	Year	1	64	25.0	4.7E-06	0.1
	poly(Week, 2)	2	186	21.2	4.9E-09	0.2
	Treatment:Year	1	54	4.8	3.4E-02	0.03

Table S2. Expected genome-wide F_{ST} values between *Daphnia magna* populations evolved in control and herbivory ponds under genetic drift. The expected F_{ST} values were estimated based on the initial allele frequencies with referred effective population size and number of generations under the scenario of genetic drift. These parameters were used to estimate the highest possible F_{ST} values under genetic drift. CI: Confidence interval.

# of generations	Effective population size	Expected F_{ST} (99% CI)	Observed F_{ST} (99% CI)
**6**	120	0.0077–0.0078	0.032–0.047
**16**	200	0.0122–0.0123	0.071–0.122

Table S3. Genes containing the SNPs that showed significant allele frequency differences between control and herbivory ponds. *P*-values were determined using the beta-binomial mixed-effects model (from R-package glmmTMB v1.1.9 (6)) with sampling time and pond block as random factors. The three SNPs that are shown in Fig. 3 are highlighted in bold.

CHR	POS	P-value	GeneID	Putative function
CH1_R	3296837	3e-09	g3733	-
CH1_R	3297877	3.9e-08	g3732	-
CH1_R	3318453	3.1e-09	Daphnia_magna_D_magna_CH1_R_000761	Cuticlin-1
CH1_R	3326249	4.8e-08	FUN_002123	-
CH1_R	3328507	3.4e-08	g3737	Ribosome-binding protein 1
CH1_R	3331142	1.2e-08	g3737	Ribosome-binding protein 1
CH1_R	3331191	1.5e-08	g3737	Ribosome-binding protein 1
CH1_R	3345507	2.9e-08	g3739	-
CH1_R	3345518	2.2e-08	g3739	-

CH1_R	3349243	3.6e-08	g3739	-
CH2_L	2021948	1.3e-08	Daphnia_magna_D_magna_CH2_L_001270	LIM/homeobox protein Lhx2
CH2_L	2233628	2.2e-09	g4458	Tachykinin-like peptides receptor 99D
CH2_L	2233630	2.3e-09	g4458	Tachykinin-like peptides receptor 99D
CH2_L	2233633	1.1e-09	g4458	Tachykinin-like peptides receptor 99D
CH2_L	4270484	8.9e-10	Daphnia_magna_D_magna_CH2_L_001057	-
CH2_L	4272659	2.4e-09	Daphnia_magna_D_magna_CH2_L_001057	Plasma kallikrein
CH2_L	4281419	2e-09	g4850	Zwei Ig domain protein zig-8
CH2_L	4281563	2.8e-09	g4850	Zwei Ig domain protein zig-8
CH2_L	4281572	3.4e-09	g4850	Zwei Ig domain protein zig-8
CH2_L	4281868	1.4e-09	g4850	Zwei Ig domain protein zig-8
CH2_L	4282099	1.9e-09	g4850	Zwei Ig domain protein zig-8
CH2_L	4289074	4.9e-08	g4850	Zwei Ig domain protein zig-8
CH2_L	4289319	2e-08	g4850	Zwei Ig domain protein zig-8
CH2_L	4289322	6.7e-09	g4850	Zwei Ig domain protein zig-8
CH2_L	4291606	1.4e-08	g4851	-
CH2_L	4315771	5.2e-08	D_magna_XINB302771	-
CH2_L	4342533	1.3e-08	g4857	-
CH2_L	4360738	3.9e-10	D_magna_XINB302782	Scavenger receptor class B member 1
CH2_L	4361537	2.1e-08	D_magna_XINB302782	Scavenger receptor class B member 1
CH2_L	4361829	2.9e-08	D_magna_XINB302782	Scavenger receptor class B member 1
CH2_L	4361831	4e-08	D_magna_XINB302782	Scavenger receptor class B member 1
CH2_L	4363525	2.7e-08	g4861	Scavenger receptor class B member 1
CH2_L	4371234	3.4e-09	g4865	Ribosome quality control complex subunit TCF25
CH2_L	4389279	1.1e-12	Daphnia_magna_D_magna_CH2_L_000302	-
CH2_L	4401148	6.1e-09	FUN_003244	Luciferin sulfotransferase
CH2_L	4411487	5.3e-08	g4871	Guanine nucleotide-binding protein G(o) subunit alpha
CH2_L	4508578	3.5e-08	D_magna_XINB302813	Fructose-1,6-bisphosphatase 1
CH2_L	4508586	1.2e-08	D_magna_XINB302813	Fructose-1,6-bisphosphatase 1
**CH2_L**	**4508591**	1.5e-09	**D_magna_XINB302813**	**Fructose-1,6-bisphosphatase 1**
CH2_L	4508598	3.9e-09	D_magna_XINB302813	Fructose-1,6-bisphosphatase 1
CH2_L	4508660	2.2e-08	D_magna_XINB302813	Fructose-1,6-bisphosphatase 1
CH2_L	4508668	2.4e-08	D_magna_XINB302813	Fructose-1,6-bisphosphatase 1
CH2_L	4523214	3.6e-09	g4890	-
CH2_L	4523278	1.8e-08	g4890	-
CH2_L	4526022	3.7e-08	D_magna_XINB302816	-
CH2_L	4527166	1.5e-09	g4892	-
CH2_L	5844097	5.5e-08	Daphnia_magna_D_magna_CH2_L_000939	Neurofilament medium polypeptide

CH2_L	5844814	2.9e-08	Daphnia_magna_D_magna_CH2_L_000939	Neurofilament medium polypeptide
CH2_L	5844847	2.9e-08	Daphnia_magna_D_magna_CH2_L_000939	Neurofilament medium polypeptide
CH2_L	5844848	5.6e-08	Daphnia_magna_D_magna_CH2_L_000939	Neurofilament medium polypeptide
CH2_L	5844892	2.3e-09	Daphnia_magna_D_magna_CH2_L_000939	Neurofilament medium polypeptide
CH2_L	5859492	8.7e-09	Daphnia_magna_D_magna_CH2_L_000934	-
CH2_L	5860627	4.4e-08	g5089	Protein obstructor-E
CH2_L	5954936	8.2e-09	g5109	Cyanophycinase
CH2_L	5964503	1e-08	Daphnia_magna_D_magna_CH2_L_000918	Urea transporter 2
CH2_L	6043839	1.6e-09	Daphnia_magna_D_magna_CH2_L_000906	Homeobox protein prospero
CH2_L	6043920	5.1e-09	Daphnia_magna_D_magna_CH2_L_000906	-
CH2_L	6043969	3.1e-08	Daphnia_magna_D_magna_CH2_L_000906	-
CH2_L	6044337	4.6e-10	Daphnia_magna_D_magna_CH2_L_000906	-
CH2_R	2356529	5.9e-09	g6043	-
CH2_R	2614664	1.2e-08	g6071	Transcription factor GATA-4
CH3_L	5712577	3.5e-08	g7823	-
CH4_L	1865544	2.1e-15	FUN_008680	Histone H4
CH5_L	2622924	6.2e-12	g12186	-
CH5_L	4457635	5e-08	g12564	Protein trachealess
**CH5_L**	**7535757**	4.3e-09	**Daphnia_magna_D_magna_CH5_L_000629**	**Ankyrin repeat domain-containing protein SOWAHC**
CH5_L	7541932	1.8e-11	g13107	-
CH5_L	7543997	2.7e-09	D_magna_XINB310443	-
CH5_L	7544021	2.8e-08	D_magna_XINB310443	-
CH5_L	7548886	4.7e-08	FUN_012241	-
CH5_L	7548914	3.2e-09	FUN_012241	-
CH5_L	7548927	2e-08	FUN_012241	-
CH5_L	7549175	3.5e-08	FUN_012241	-
CH5_L	7549238	5.4e-09	FUN_012241	-
CH5_L	7553410	3.1e-08	g13108	N6-adenosine-methyltransferase TMT1A
CH5_L	7649296	3.4e-08	g13127	-
CH5_L	7649334	3e-09	g13127	-
CH5_L	7649349	1.1e-08	g13127	-
CH6	8635704	3.4e-09	D_magna_XINB301668	Tetraspanin-3
CH6	8650501	1.3e-08	g15669	Kinesin-like protein KIF3A
CH7_L	1927186	3.1e-08	g16459	Serine/threonine-protein kinase N
CH7_R	306771	2.9e-08	g17612	-
CH7_R	312466	2.2e-08	g17615	-
CH7_R	450106	3.4e-08	Daphnia_magna_D_magna_CH7_R_000089	-
CH7_R	450870	2e-08	g17671	-
CH7_R	450882	1e-09	g17671	-

CH7_R	455399	6.9e-10	g17673	-
CH7_R	455400	8e-10	g17673	-
CH7_R	460581	3e-09	Daphnia_magna_D_magna_CH7_R_000821	-
CH7_R	461672	4.6e-08	g17675	Tetraspanin-2A
**CH7_R**	**461798**	2.7e-13	**g17675**	**Tetraspanin-2A**
CH7_R	461995	4.5e-09	g17675	Tetraspanin-2A
CH7_R	462165	8.2e-12	g17675	Tetraspanin-2A
CH7_R	462174	2.3e-12	g17675	Tetraspanin-2A
CH7_R	462178	4.4e-13	g17675	Tetraspanin-2A
CH7_R	462191	3.5e-12	g17675	Tetraspanin-2A
CH7_R	462202	3.3e-10	g17675	Tetraspanin-2A
CH10_L	146278	1.6e-09	D_magna_XINB321143	-
CH10_L	147497	3.2e-08	g43	-
CH10_L	147500	5.7e-08	g43	-
CH10_L	147501	3.1e-08	g43	-
CH10_L	151992	3.6e-08	g44	Histone deacetylase 6
**CH10_L**	**152191**	**5.2e-08**	**g44**	**Histone deacetylase 6**
CH10_L	2610115	4e-08	g546	Serine/threonine-protein kinase 26
CH10_L	974213	2.8e-10	g263	-

SI References

1. A. Malacrinò *et al.*, Induced responses contribute to rapid adaptation of *Spirodela polyrhiza* to herbivory by *Lymnaea stagnalis*. *Commun Biol* **7**, 81 (2024).
2. H. Utermöhl, Neue Wege in der quantitativen Erfassung des Plankton.(Mit besonderer Berücksichtigung des Ultraplanktons.). *SIL Proceedings* **5**, 567-596 (1931).
3. D. E. Wood, J. Lu, B. Langmead, Improved metagenomic analysis with Kraken 2. *Genome Biol* **20** (2019).
4. A. Thivolle, M. Paljakka, D. Ebert, P. D. Fields, The genome of *Pasteuria ramosa* reveals a high turnover rate of collagen-like genes. *bioRxiv*, 2024.2002.2009.579640 (2024).
5. M. Fredericksen, P. D. Fields, L. Du Pasquier, V. Ricci, D. Ebert, QTL study reveals candidate genes underlying host resistance in a Red Queen model system. *PLoS Genet.* **19**, e1010570 (2023).
6. M. E. Brooks *et al.*, glmmTMB balances speed and flexibility among packages for zero-inflated generalized linear mixed modeling. *R J* **9**, 378-400 (2017).
